# The interactions of SARS-CoV-2 with cocirculating pathogens: Epidemiological implications and current knowledge gaps

**DOI:** 10.1371/journal.ppat.1011167

**Published:** 2023-03-08

**Authors:** Anabelle Wong, Laura Andrea Barrero Guevara, Elizabeth Goult, Michael Briga, Sarah C. Kramer, Aleksandra Kovacevic, Lulla Opatowski, Matthieu Domenech de Cellès

**Affiliations:** 1 Infectious Disease Epidemiology group, Max Planck Institute for Infection Biology, Berlin, Germany; 2 Institute of Public Health, Charité–Universitätsmedizin Berlin, Berlin, Germany; 3 Epidemiology and Modelling of Antibiotic Evasion, Institut Pasteur, Université Paris Cité, Paris, France; 4 Anti-infective Evasion and Pharmacoepidemiology Team, CESP, Université Paris-Saclay, Université de Versailles Saint-Quentin-en-Yvelines, INSERM U1018 Montigny-le-Bretonneux, France; University of Alberta, CANADA

## Abstract

Despite the availability of effective vaccines, the persistence of severe acute respiratory syndrome coronavirus 2 (SARS-CoV-2) suggests that cocirculation with other pathogens and resulting multiepidemics (of, for example, COVID-19 and influenza) may become increasingly frequent. To better forecast and control the risk of such multiepidemics, it is essential to elucidate the potential interactions of SARS-CoV-2 with other pathogens; these interactions, however, remain poorly defined. Here, we aimed to review the current body of evidence about SARS-CoV-2 interactions. Our review is structured in four parts. To study pathogen interactions in a systematic and comprehensive way, we first developed a general framework to capture their major components: sign (either negative for antagonistic interactions or positive for synergistic interactions), strength (i.e., magnitude of the interaction), symmetry (describing whether the interaction depends on the order of infection of interacting pathogens), duration (describing whether the interaction is short-lived or long-lived), and mechanism (e.g., whether interaction modifies susceptibility to infection, transmissibility of infection, or severity of disease). Second, we reviewed the experimental evidence from animal models about SARS-CoV-2 interactions. Of the 14 studies identified, 11 focused on the outcomes of coinfection with nonattenuated influenza A viruses (IAVs), and 3 with other pathogens. The 11 studies on IAV used different designs and animal models (ferrets, hamsters, and mice) but generally demonstrated that coinfection increased disease severity compared with either monoinfection. By contrast, the effect of coinfection on the viral load of either virus was variable and inconsistent across studies. Third, we reviewed the epidemiological evidence about SARS-CoV-2 interactions in human populations. Although numerous studies were identified, only a few were specifically designed to infer interaction, and many were prone to multiple biases, including confounding. Nevertheless, their results suggested that influenza and pneumococcal conjugate vaccinations were associated with a reduced risk of SARS-CoV-2 infection. Finally, fourth, we formulated simple transmission models of SARS-CoV-2 cocirculation with an epidemic viral pathogen or an endemic bacterial pathogen, showing how they can naturally incorporate the proposed framework. More generally, we argue that such models, when designed with an integrative and multidisciplinary perspective, will be invaluable tools to resolve the substantial uncertainties that remain about SARS-CoV-2 interactions.

## 1. Introduction

As of August 2022, the pandemic of coronavirus disease 2019 (COVID-19)—caused by the novel severe acute respiratory syndrome coronavirus 2 (SARS-CoV-2)—has resulted in at least 598 million cases and 6.4 million deaths worldwide [1]. Despite the implementation of stringent control measures and the increasing rollout of effective vaccines in many locations, the persistent circulation of SARS-CoV-2 suggests the infeasibility of elimination and the gradual transition to endemic or seasonal epidemic dynamics [2]. Hence, cocirculation of SARS-CoV-2 with other pathogens may become increasingly frequent and cause multiple simultaneous epidemics of, for example, COVID-19 and influenza [3].

**Fig 1:**
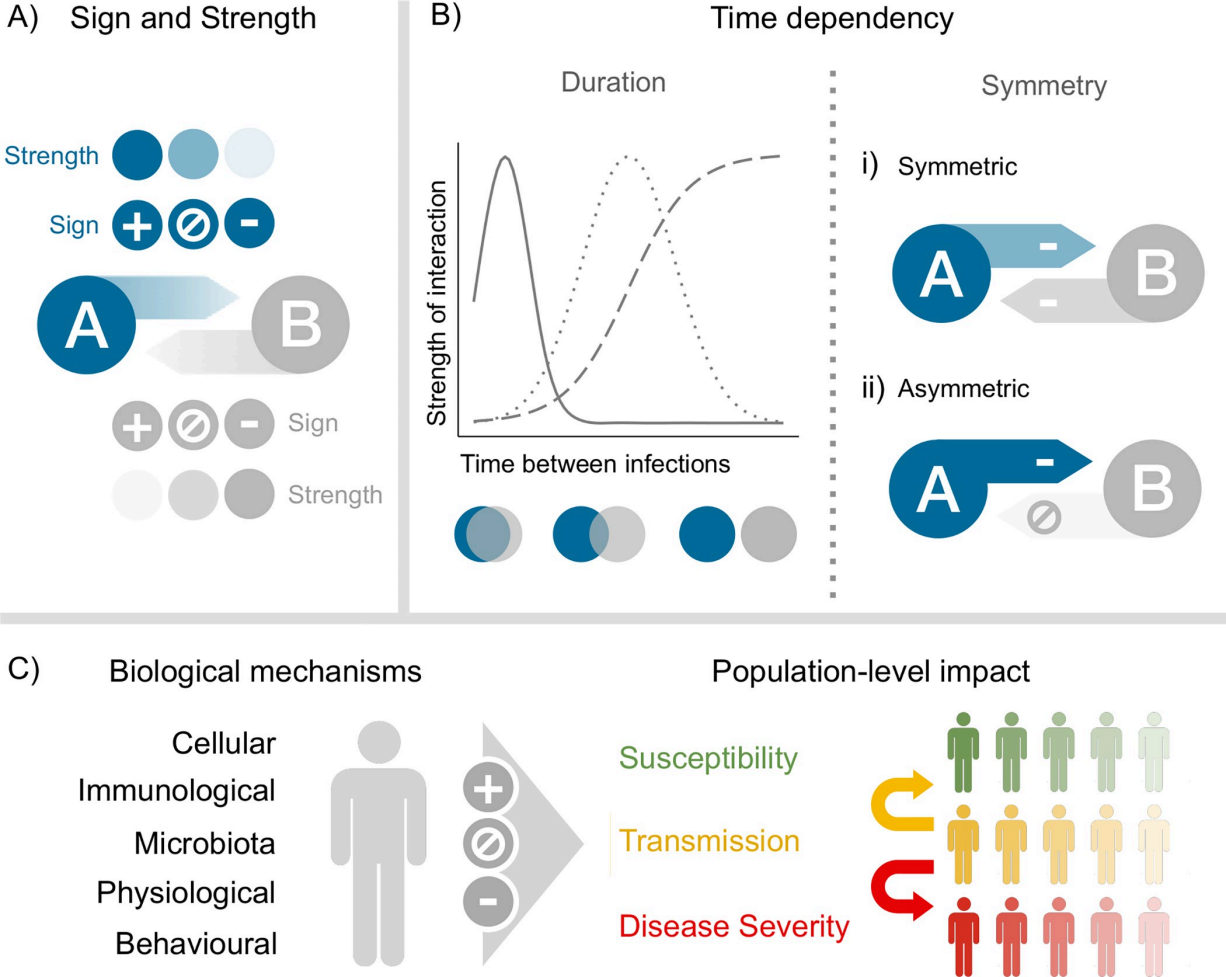
A conceptual framework to study pathogen interactions. For a given pair of pathogens, interaction can be characterized by its sign and strength (panel A), which, in turn, depend on the time interval between infections (duration of interaction) and on the sequence of infection (symmetry of interaction) (panel B). Examples include negative, symmetric interactions (as in the case of influenza B virus Victoria lineage and Yamagata lineage [15]) and negative, asymmetric interactions (as in the case of rhinovirus and influenza A virus or respiratory syncytial virus [18]). Interaction can be caused by different biological mechanisms (panel C), which determine its positive or negative effects on susceptibility to infection, transmission (transmissibility and duration of infection), or disease severity at the individual level and, in turn, its impact at the population level.

Interaction—i.e., the ability of one pathogen to alter the risk of infection or disease caused by another pathogen (Fig 1)—is an essential aspect to forecast the dynamics of cocirculating infectious diseases. From a public health perspective, interactions may significantly aggravate the incidence of infection and the disease burden, as demonstrated for immunosuppressive viruses like measles [4] and human immunodeficiency virus (HIV) [5]. Another interesting, yet understudied public health implication of interactions is the possibility of off-target effects of vaccines on nontarget pathogens, as suggested for influenza vaccines [6,7]. However, despite their potentially large relevance to SARS-CoV-2 epidemiology and COVID-19 control measures, the interactions of SARS-CoV-2 with other pathogens remain poorly defined.

Here, we aimed to review the current body of evidence about the interactions of SARS-CoV-2 with cocirculating pathogens. We first present a general framework to capture the complexities of interactions and study them in a systematic and comprehensive way. Using this framework, we then review the results of published experimental and epidemiological studies. Finally, we formulate simple transmission models incorporating the proposed framework to illustrate the potential population-level impact of SARS-CoV-2 interactions.

## 2. Dissecting pathogen interactions: sign and strength, timing, and mechanisms

Multiple elements are needed to fully characterize pathogen interactions, making their general study complex. To study interactions in a systematic and comprehensive way, we propose a conceptual framework—depicted schematically in Fig 1—that incorporates three essential components of interaction, detailed below.

### 2.1. Sign and strength of interaction

The first dimension of this framework is the sign and strength of interaction. Here, we define the sign of interaction as positive in synergistic interactions (where a first pathogen increases the risk of infection or disease of a second pathogen) and negative in antagonistic interactions (where the risk is decreased), and we refer to strength as the magnitude of the effect on a given parameter exerted by one pathogen on another.

An example of negative interaction exists between influenza A virus (IAV) and human respiratory syncytial virus (RSV), for which experimental studies have shown that a recent IAV infection inhibits the growth of RSV in ferrets [8] and in mice [9]. By contrast, IAV interacts positively with *Streptococcus pneumoniae* (the pneumococcus) by promoting bacterial growth [10,11]. This illustrates that interaction is pathogen-specific and cannot be easily extrapolated to other pathogen systems.

### 2.2. Time dependency of interaction

The second dimension of our proposed framework is time dependency: Both the time between infections and the sequence of infection can affect the sign and strength of an interaction.

#### Duration of interaction and time between infections

Due to the kinetics of cellular and humoral immune responses following respiratory infections [12–14], the strength of interaction can change with time between infections. For example, primary IAV infection prevented subsequent RSV infection in ferrets when exposed 3 days later, but the protection disappeared as the time between IAV and RSV challenges increased to 11 days [8]. Such short-lived negative interaction was also observed between influenza B virus Victoria lineage (B/Vic) and Yamagata lineage (B/Yam) [15]. Interaction can be long-lived if it is mediated by immune memory. For example, measles infection can partially erase previously acquired immunity to other pathogens, causing “immune amnesia” [16]. Childhood exposures to a given IAV subtype can cause long-lasting immunological bias that shapes the individual’s subsequent risk for influenza infection [17].

#### Symmetry of interaction and sequence of infection

The sequence of infection can also affect the interaction, as evidenced by the asymmetric effects found in previous studies. For example, prior infection with IAV or RSV hindered rhinovirus (RV) replication, but prior RV infection did not interfere with IAV and RSV replication in human airway epithelium [18]. While IAV infection predisposed individuals to pneumococcal colonization and infection [19–21] and led to more severe disease [22], evidence from animal and human challenge studies demonstrated that prior pneumococcal colonization did not lead to more severe disease [20,23,24] but might have had a protective effect against viral replication [24,25] upon subsequent IAV challenge. Interestingly, this effect might depend on the density of pneumococcal colonization [20,23,24].

By contrast, when a negative interaction is symmetric between two pathogens, whichever of the two pathogens is the first to infect can inhibit subsequent infection by the other pathogen—as in the case of influenza B lineages [15].

### 2.3. Biological mechanisms and population-level impact of interaction

The third dimension in our framework is the mechanism of interaction: Interaction can be caused by different biological mechanisms, which determine its positive or negative effects on susceptibility to infection, characteristics of infection (such as transmissibility and duration), or disease severity at the individual level and, in turn, its impact at the population level (Fig 1C).

#### Biological mechanisms

Examples of biological mechanisms of pathogen interaction include intracellular and physiological changes and effects on the immune response, on the respiratory microbiota, and on host behaviors. A pathogen can induce changes to the host cells that are beneficial or detrimental to another pathogen. For example, it has been shown that RSV and human parainfluenza virus 3 (HPIV-3) increase the expression of receptors for *Haemophilus influenzae* and the pneumococcus binding in bronchial epithelial cells [26]. In both cases, changes in cellular expression may lead to a positive interaction. A pathogen can cause changes to the host’s immune profile (e.g., depletion of CD4+ T cells by HIV [5], increased interferon response by IAV [9]), facilitating or hindering infection with a second pathogen. Moreover, a pathogen can change the physiological environment to potentiate a secondary infection by another pathogen. For example, the replication of IAV in the respiratory epithelium reduces mucociliary clearance and damages epithelial cells, resulting in enhanced attachment and invasion of the pneumococcus [21]. Changes in the respiratory tract microbiota by an infection can lead to the acquisition of a new pathogen or to overgrowth and invasion of an already present pathogen [27–29]. Lastly, changes in host behaviors caused by infection with a first pathogen can affect the risk of subsequent infection with another pathogen, even in the absence of within-host interaction between the two. Examples include self-isolation to reduce the spread of disease in humans and reduced social contacts between infected animals [30,31].

Additionally, the existence of other interaction mechanisms is suggested by a recent study, which demonstrated that coinfection with IAV and RSV could lead to the formation of hybrid viral particles with altered antigenicity and expanded cell tropism [32].

#### Population-level impact

The biological mechanisms outlined above may affect population-level dynamics through their effects on different epidemiological parameters: susceptibility to infection, transmission of infection (characterized by the transmissibility and the duration of infection), and disease severity. Hence, estimating how these parameters vary in individuals coinfected or previously infected with a potential interacting pathogen can provide quantitative evidence for different mechanisms of interaction. Of note, multiple biological mechanisms can affect the same epidemiological parameter; conversely, the same biological mechanism can affect multiple epidemiological parameters. For example, IAV-induced epithelial damage and dampened pneumococcal clearance increase host susceptibility to the pneumococcus and disease severity in coinfection, as suggested by historical pandemics [33], demonstrated in experimental studies [19], and inferred from mechanistic modeling of epidemiological time-series [34,35]. The effect of interaction on transmission is more difficult to measure, as it is determined not only by the susceptibility of the exposed and the transmissibility of the infected, but also by the contact patterns between the two [36]. However, this effect can be approximated with animal models [37–39] or estimated with mathematical modeling based on epidemiological data [36]. Of note, as shown by the decline in various respiratory infections following the nonpharmaceutical interventions in the COVID-19 pandemic [36,40–43], transmission can be changed substantially by host behaviors.

## 3. Review of evidence on SARS-CoV-2 interactions

### 3.1. Experimental evidence from animal models

Having proposed a framework to study interactions, we now review experimental studies on coinfections with SARS-CoV-2 in animal models. As of August 22, 2022, we identified 14 publications [44–57]. We first review the 11 studies that focused on SARS-CoV-2 and nonattenuated IAV.

#### Experimental studies of coinfection with SARS-CoV-2 and nonattenuated IAV

**Fig 2:**
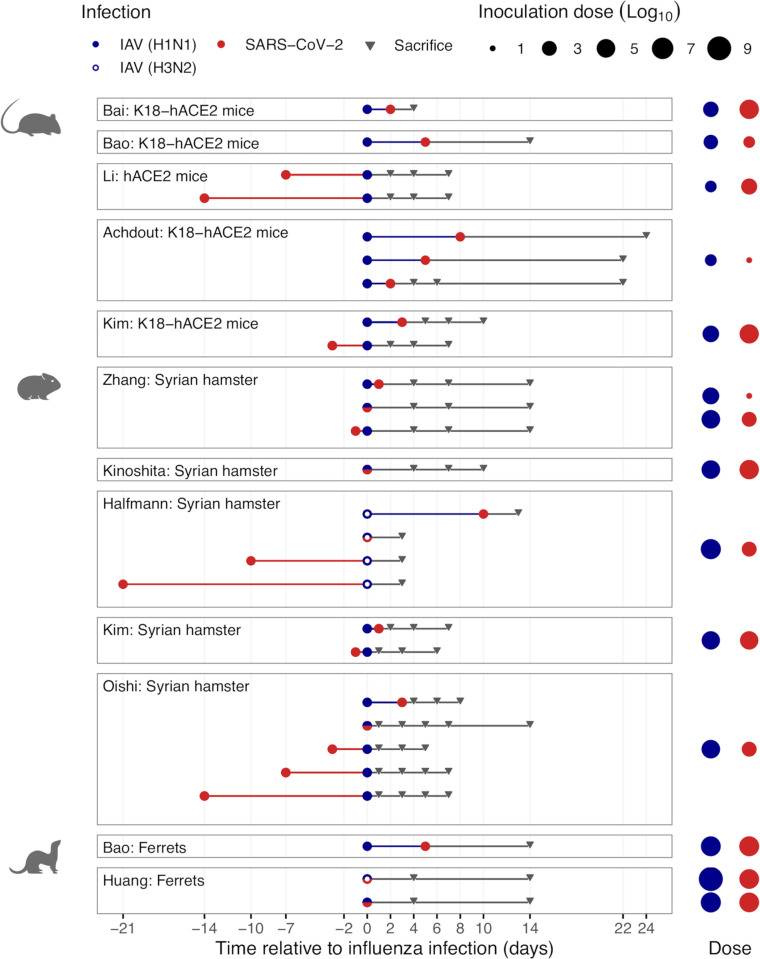
Experimental designs of animal studies assessing the interaction between SARS-CoV-2 and influenza A virus (IAV). [44–54]. The protocols of the experiments in mice, hamsters, and ferrets were summarized to allow comparison across studies. Red points represent the time point for infection with SARS-CoV-2 and blue points for infection with IAV (filled points represent infection with IAV (H1N1) and empty points with IAV (H3N2)). Triangles represent the time at which samples from sacrificed animals were taken. The data from every study were extracted from the text, the tables, or the figures; all the corresponding values were checked and are available in S1 and S2 Tables.

As shown in Fig 2, three different animal models were used (ferrets, hamsters, and mice), and the experimental designs varied substantially across the 11 studies, particularly in the sequence of infection, the time between infections (range: 0 to 21 days), and the follow-up duration (range: 3 to 24 days since first infection, 2 to 20 days since the second infection). Nine studies [44–48,50–53] examined coinfections with IAV preceding SARS-CoV-2, six [47,49–53] with SARS-CoV-2 preceding IAV, and five with simultaneous infections [47,48,50,52,54]. Of note, only three studies [47,50,52] compared all three infection sequences, and only four studies [46,49,50,52] compared different times between infections. Furthermore, the studies also varied widely in the inoculation dose (IAV range: 8 × 10^1^ to 1.3 × 10^9^ PFU; SARS-CoV-2 range: 1 × 10^1^ to 7 × 10^5^ PFU), with a single study [47] evaluating the effect of different doses. The studies used different IAV subtypes (H1N1 [44–49,51–54] and H3N2 [50,54]) and SARS-CoV-2 lineages (A [49,51,52,54], B [44,45,47,53], B.1 [46,50], and B1.1 [48]), as well as different strains within subtypes and lineages. Finally, only one study compared the effects of IAV (H1N1) and IAV (H3N2) [54]. Due to the limited number of studies and the large heterogeneity across them, we compare the results for SARS-CoV-2 and IAV (H1N1) coinfection only qualitatively.

**Fig 3:**
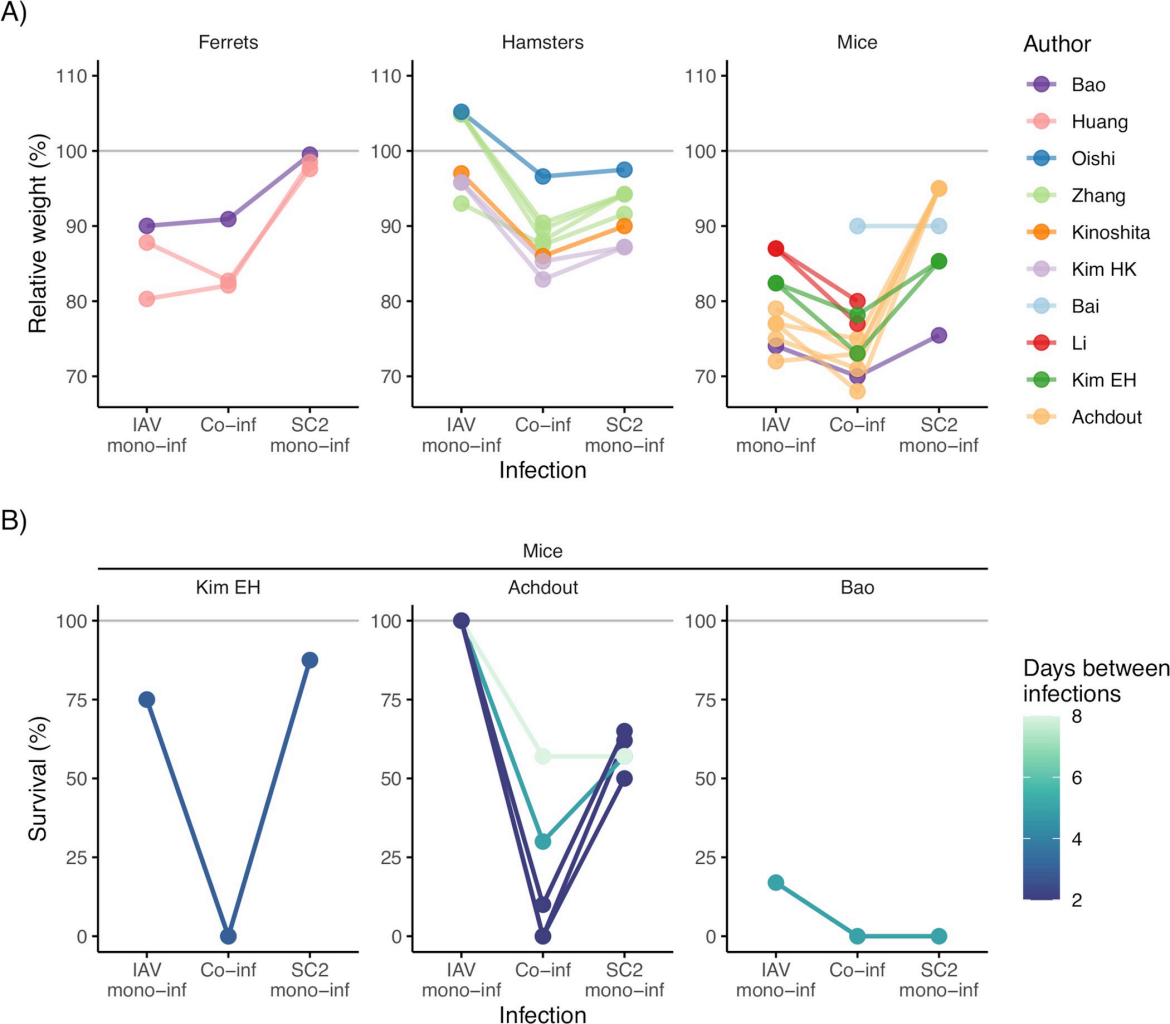
Summary results from animal studies assessing the effect of coinfection with SARS-CoV-2 and influenza A virus (IAV) on disease severity. [44–49,51–54]. In panel A, the *y*-axis values represent the weight relative to baseline, calculated when the maximal weight loss was observed (or, if the animals did not lose weight, when the maximum weight gain was observed), and colors represent different studies. In panel B, the *y*-axis values represent the fraction of animals alive at the end of the experiment, and colors represent the times between infections. The data from every study were extracted from the text, the tables, or the figures; all the corresponding values were checked and are available in S1 and S2 Tables.

As shown in Fig 3A, the severity of monoinfection with either IAV or SARS-CoV-2 differed between animal models. In ferrets, monoinfection with IAV, but not with SARS-CoV-2, resulted in weight loss, while the opposite was observed in hamsters. In mice, however, both monoinfections generally caused weight loss. Also, unlike the hamster and ferret models, mice can develop severe COVID-19 and die, so this model was used in all studies that analyzed survival (Fig 3B). On the whole, these results agree with earlier evidence of the advantages and limitations of different animal models for *in vivo* research on IAV and SARS-CoV-2 [58,59].

In all but one study, the effect of coinfection on disease severity was quantified by tracking changes in the animals’ body weight. In mice and, to a lesser extent, in hamsters, animals coinfected suffered a higher maximal weight loss than animals monoinfected with either IAV or SARS-CoV-2 (Fig 3A, S1 Table). In ferrets, however, the maximum weight loss after coinfection was relatively comparable to that after IAV monoinfection. In keeping with the results based on weight loss, the three studies that measured survival (all using the mice model) found that coinfected animals either suffered higher mortality [46,51] or died faster [45] than monoinfected animals (Fig 3B, S1 Table).

**Fig 4:**
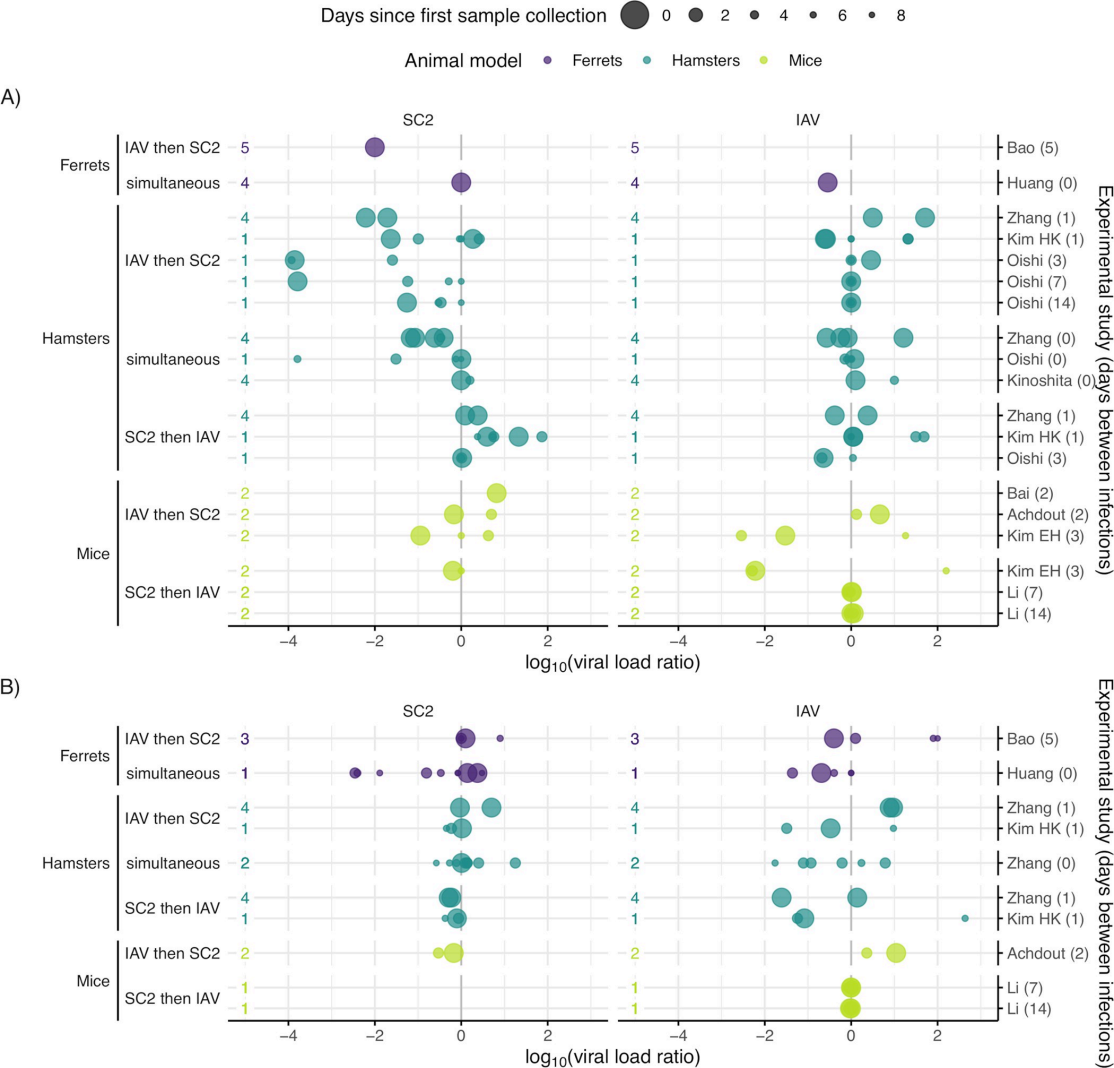
Summary results from animal studies assessing the effect of coinfection with SARS-CoV-2 and influenza A virus (IAV) on viral loads. [44–49,51–54]. The *x*-axis values represent the log_10_ ratio of the viral load of SARS-CoV-2 (left panels) or IAV (right panels) during coinfection to that during monoinfection, in either the lower respiratory tract (panel A) or the upper respiratory tract (panel B). Every line represents the results of one experiment, whose settings are summarized by four elements: (1) the sequence of infection, indicated by the text on the left *y*-axis; (2) the time interval between infections, indicated by the number between parentheses on the right *y*-axis; (3) the time of first sample collection (relative to the time of last infection), indicated by the colored number on the left *y*-axis; and (4) the times of subsequent sample collection (relative to the time of first sample collection), indicated by the size of the points on the lines. For example, in the study by Kim EH (panel A, mice), the sequence of infection was IAV then SARS-CoV-2 with 3 days separating the infections; the first sample was then collected 2 days after SARS-CoV-2 infection, and then 2 and 5 days after that first sample. The data from every study were extracted from the text, the tables, or the figures; all the corresponding values were checked and are available in S1 and S2 Tables.

In contrast to the relatively consistent results on disease severity, the effect of coinfection on viral load—quantified as the ratio of viral load during coinfection to that during monoinfection—was more heterogeneous across studies (Fig 4, S2 Table). In addition to the sources of heterogeneity outlined above, the studies varied in the technique used to quantify viral load (either reverse transcription quantitative polymerase chain reaction [RT-qPCR], plaque-based, or median tissue culture infectious dose [TCID50] assays) and in the sample type (swabs or tissue) and location (lower respiratory tract [LRT] or upper respiratory tract [URT]). These differences may affect the inferred sign and strength of interaction: For example, the load of infectious viruses—which only plaque-based or TCID50 assays can quantify—in the URT is likely a more relevant proxy of transmissibility [60] but was measured in only six studies [46,47,50,52–54]. Overall, the effect size spanned six orders of magnitude and depended on the location of the body compartment sampled. In the LRT, the viral load of SARS-CoV-2 was generally reduced by preceding or simultaneous infection with IAV but increased by subsequent infection with IAV in hamsters (Fig 4A, left panel). The effect was more variable in mice and inconclusive in ferrets because of a low number of studies. On the other hand, there was no obvious pattern in the viral load of IAV, regardless of infection order (Fig 4A, right panel). In the URT, fewer studies assessed the effect of coinfection on viral load and their results were inconsistent (Fig 4B).

Of note, several studies suggested time dependencies in coinfection outcomes. First, the maximum weight loss was typically observed 7 to 12 days post-infection ([45,46]; S1 Table), so studies with shorter follow-ups could underestimate disease severity. Second, shorter times between infections were found to increase disease severity in two studies [46,49] (Fig 3B, S1 Table) and the effect on viral load in one study [52].

In conclusion, despite large heterogeneity and inconsistencies across the studies reviewed, the collective evidence from animal models shows that coinfection with IAV and SARS-CoV-2 causes more severe disease than monoinfection with either virus. Despite having clinical relevance, these results do not necessarily demonstrate a positive interaction. This is because the disease severity endpoints in all studies were nonspecific, making it difficult to hypothesize the expected disease severity resulting from the mere co-occurrence of two independent infections that do not interact. For example: if virus A monoinfection causes 10% mortality and virus B monoinfection 20% mortality, what would be the expected mortality of coinfection if the two viruses do not interact? Although it has been proposed that synergism is evidenced whenever the severity of coinfection exceeds the maximal severity of monoinfection (20% in our example) [61], this definition seems unsatisfactory when both pathogens cause disease. A way to circumvent this attribution problem would be to identify virus-specific endpoints predictive of disease severity. Despite the availability of such endpoints to assess the effect of coinfection on viral load, the collective evidence was inconclusive. A generally robust finding was that preceding or simultaneous infection with IAV reduced the viral load of SARS-CoV-2 in the LRT. However, only a few studies measured the viral load in the URT, which is likely a more relevant proxy of transmissibility [60]. Therefore, further studies will be needed to demonstrate the existence of interactions affecting susceptibility to, or transmissibility of, infection. In the design of such studies, we argue that the strength of evidence could be increased by varying the infectious dose (to better assess the strength of interaction) and the infection order (to assess the symmetry of interaction) and by considering different animal models (to check for robustness).

#### Experimental studies of coinfection with SARS-CoV-2 and other pathogens

In addition to the previous studies, we identified three experimental studies on SARS-CoV-2 coinfection [55–57]. One study found that administering live attenuated influenza A vaccine 3 days before SARS-CoV-2 infection reduced SARS-CoV-2 viral load in ferrets [55]. The second study observed that SARS-CoV-2 infection after, but not before, pneumococcal infection, increased the viral and bacterial loads, worsening disease severity and survival [57]. In contrast, the third study found that chronic infection with *Mycobacterium tuberculosis* inhibited SARS-CoV-2 viral load, decreasing disease severity [56].

### 3.2. Epidemiological evidence

Although experimental studies using animal models can inform some of the components required to characterize pathogen interactions (Fig 1), they are insufficient in predicting the public health impact of interaction in humans, for at least two reasons. First, animal models cannot fully recapitulate the biology of infection in humans, as illustrated by the ongoing search for an appropriate animal model representative of severe COVID-19 disease in humans [58]. Second, animal experimental studies may be underpowered to estimate the relative risk of infection or severe disease in co- versus monoinfected individuals. Hence, epidemiological studies remain indispensable to assess the significance of interaction in human populations. We, therefore, reviewed the literature on SARS-CoV-2 and coinfections in human populations. The identified studies are classified into three categories: (1) studies that were based on coinfection prevalence; (2) studies that examined the association between non-COVID vaccines and COVID-19; and (3) studies that examined the association between prior respiratory infections and COVID-19.

#### Studies based on the detection of SARS-CoV-2 coinfections

Studies based on the detection of SARS-CoV-2 coinfections attempted to answer two research questions: (1) whether coinfection with other pathogens change the severity of COVID-19, or (2) whether the detection of other pathogens was associated with a change in SARS-CoV-2 detection.

Four meta-analyses, covering a total of 95 studies that reported mortality outcomes, addressed the first question. The first meta-analysis included only four studies, with large heterogeneity [62]. The second meta-analysis (which included 12 studies, of which 9 reported mortality) estimated reduced mortality in patients coinfected with influenza from studies in China, (OR = 0.51, 95% CI: 0.39 to 0.68, I^2^ = 26.5%) but increased mortality from studies outside China (OR = 1.56, 95% CI: 1.12 to 2.19, I^2^ = 1%) [63]. The two other meta-analyses reported higher mortality in SARS-CoV-2 coinfections compared with SARS-CoV-2 monoinfections. However, one of them (which included 59 studies, of which 10 reported case fatality) did not provide information about the infection order [64]; the other (which included 118 studies, of which 84 reported mortality) provided separate estimates for when other respiratory pathogens were detected at the time of SARS-CoV-2 detection (OR = 2.84, 95% CI: 1.42 to 5.66) or after (OR = 3.54, 95% CI: 1.46 to 8.58), but pooled estimates for different age groups, healthcare settings (ICU and non-ICU), and pathogens (bacterial, viral and fungal) [65]. In general, all these studies require cautious interpretation, because confounders (such as comorbidities) may bias estimation.

Two studies used a test-negative design to address the second question, by comparing the prevalence of SARS-CoV-2 infection (or other respiratory pathogen infection) in groups infected versus uninfected with another pathogen (or with SARS-CoV-2) [66,67]. The first did not find statistically significant differences in the proportion positive for other respiratory pathogens (including influenza) between patients negative and positive for SARS-CoV-2 [66], while the other reported a 58% decrease in the risk of testing positive for SARS-CoV-2 among influenza-positive cases [67]. However, this approach can be inappropriate for two reasons. First, the prevalence of coinfections was likely underestimated due to the prescription of empirical antibiotic treatment prior to microbiological investigation [68,69] and due to diagnostic strategies favoring SARS-CoV-2 diagnosis [70]. Moreover, when simultaneous testing of multiple pathogens is limited, epidemics of cocirculating pathogens may artificially decrease the positivity fraction of SARS-CoV-2 [71]. Second, a less appreciated, but more essential problem of test-negative designs is that they can systematically underestimate the strength of interaction and frequently infer the wrong sign of interaction for seasonal and emerging respiratory viruses [72]. These issues caution against simple and seemingly intuitive measures of pathogen interactions based on coinfection prevalence data, echoing earlier studies in infectious disease ecology and epidemiology [73–75].

#### Studies examining the association between non-COVID vaccination history and COVID-19

Since interacting pathogens form polymicrobial systems, interventions against any pathogen in such systems may theoretically affect the others. For example, if there is a positive interaction between a vaccine-preventable respiratory pathogen (e.g., IAV or the pneumococcus) and SARS-CoV-2, one may expect, with all else being equal, SARS-CoV-2-related outcomes to be higher in unvaccinated individuals. A systematic review [76] and two meta-analyses [77,78] have summarized a total of 30 articles on observational studies investigating the association of influenza vaccine and SARS-CoV-2 infections and outcomes. While the earlier systematic review (which included 12 studies) indicated that only some studies reported significant inverse associations between influenza vaccination and SARS-CoV-2-related outcomes [76], the later meta-analyses (which included 16 [77] and 23 studies [78], respectively) found a significantly lower risk of SARS-CoV-2 infection associated with influenza vaccination (OR: 0.86, 95% CI: 0.81 to 0.91 [77]; OR: 0.83, 95% CI: 0.76 to 0.90 [78]).

In contrast to influenza vaccines, we found no systematic review that examined the association between pneumococcal conjugate vaccines (PCVs) or pneumococcal polysaccharides vaccines (PPSVs) and SARS-CoV-2 outcomes. Based on a literature review, we identified four studies—2 on PCV and PPSV [79,80], 1 on PPSV only [81], and 1 on PCV only [82] (S3 Table). All three studies involving PPSV did not find conclusive evidence for an association between PPSV history and SARS-CoV-2-related outcomes [79–81]. PCV was associated with protection against COVID-19 infection, hospitalization, and mortality among older adults in one cohort study [80], and against symptoms among SARS-CoV-2-infected children in another cohort study [82]. Although inconclusive, the association estimated in a case–control study [79] was consistent with that in the two cohort studies.

Findings from vaccine impact studies must be interpreted with caution when attempting to infer pathogen interactions. First, although numerous studies attempted to estimate the effect of various vaccines on COVID-19 outcomes, few accounted for healthy user bias, a common form of selection bias whereby more active health-seeking behaviors can be a source of confounding [83]. As acknowledged by [84] and [85], this is often a limitation in observational studies, as influenza vaccination is voluntary [85–88]. Second, even when epidemiological studies adopting more robust study designs (e.g., prospective cohort) and inference methods (e.g., Cox model with inverse propensity weighting) show that non-SARS-CoV-2 vaccines confer protection against SARS-CoV-2 [80], one cannot distinguish if such protection stems from hindering the positive interaction between two pathogens, or from the direct effect of the vaccine on SARS-CoV-2—for example, via nonspecific immune responses such as trained innate immunity [89].

#### Studies examining the association between prior respiratory infections and COVID-19

Four observational studies reported the association between prior respiratory infections and COVID-19-related outcomes [90–93] (S4 Table). Prior influenza infection was reported to be associated with increased COVID-19 susceptibility (OR: 3.07, 95% CI: 1.61 to 5.85 for 1 to 14 days prior, OR 1.91, 95% CI: 1.54 to 2.37 for 1 to 90 days prior) and severity (OR: 3.64, 95% CI: 1.55 to 9.21 for 1 to 14 days prior, OR: 3.59, 95% CI: 1.42 to 9.05 for 1 to 30 days prior) in a case–control study [90]. This evidence, suggestive of a positive interaction between influenza and SARS-CoV-2, is consistent with the findings from a mathematical modeling study [94]. Although a retrospective cohort study reported that prior infection with endemic human coronaviruses (hCoVs) was associated with protection against COVID-19-related ICU admission (OR: 0.1, 95% CI: 0.1 to 0.9) [91], a case–control study on serum samples from hospitalized COVID-19 patients found that hCoVs antibodies were not associated with protection against SARS-CoV-2 infections nor hospitalizations [92]. Regarding the impact of upper respiratory infections (URIs), a retrospective cohort study found lower risk (OR: 0.76, 95% CI: 0.75, 0.77) of testing positive for SARS-CoV-2 among individuals with URI diagnosed in the preceding year [93], while a case–control study found higher risk among individuals diagnosed with URI in the preceding 1 to 14 days (OR: 6.95, 95% CI: 6.38 to 7.58) and 1 to 90 days (OR: 2.70, 95% CI: 2.55 to 2.86) [90]. This discrepancy may be explained by the different URI definitions and time frames for exposure measurement, in addition to different study designs and included confounders. Because these studies provided information about the infection timeline, they offered stronger evidence to infer pathogen interactions than studies based on coinfection prevalence, and also more direct evidence than studies examining the association between non-COVID vaccines and COVID-19. Nevertheless, one should beware of how misclassification of exposure and imperfect control for confounding can limit such study designs in inferring pathogen interactions.

In summary, the evidence available from human population health data indicates that coinfection prevalence is largely variable, that influenza vaccines and PCVs may be associated with reduced risk of SARS-CoV-2, and that earlier influenza infection may be associated with increased risk of SARS-CoV-2 infection and disease severity. However, our review also highlighted the limitations in the current epidemiological literature, as many studies were prone to multiple biases, including confounding, and only very few [90–94] were designed to infer interaction.

## 4. The need for transmission models to study epidemiological interactions

As reviewed above, the results of epidemiological studies can be difficult to interpret and their designs insufficient to characterize all the components of interactions (Fig 1). Arguably, more integrated approaches are therefore needed to capture the complexities described above and to determine how individual-level mechanisms of interaction translate into population-level dynamics of infection or disease.

**Fig 5:**
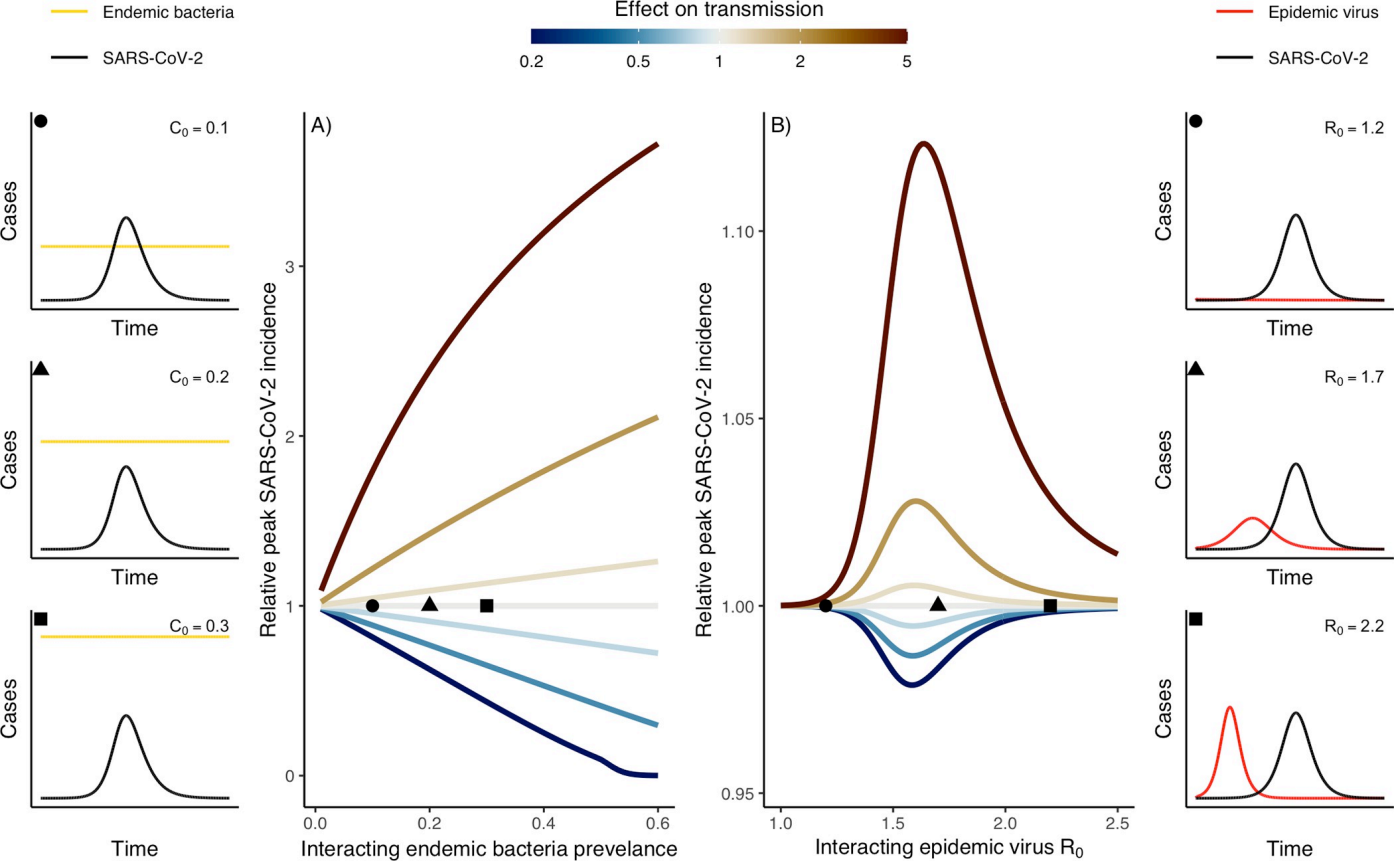
Predicted impact of interaction on SARS-CoV-2 dynamics from mathematical models of SARS-CoV-2 interaction. Panel A: Interaction with colonizing bacteria (e.g., *Streptococcus pneumoniae*). Panel B: Interaction with a seasonal virus (e.g., influenza A virus). Insets represent three example simulations for each of the two models, varying the prevalence of bacterial colonization (*C*_0_) and the basic reproduction number (*R*_0_) of the interacting virus (the circle, triangle, and square symbols indicate the corresponding parameter values in the main figures). Note, the vertical axes are on different scales, showing the more pronounced impact of interactions with endemic colonizing bacteria. The data presented are primary data, generated from illustrative models designed for the purpose of this review; full model details are included in S1 Appendix.

Mathematical models of transmission offer a powerful and economical tool to study infectious disease dynamics [95]. To study pathogen interactions, such models can be formulated to incorporate biologically explicit mechanisms of interaction (in addition to the other elements of the framework proposed above) and predict their potentially nonlinear effects on transmission dynamics [96]. By design, these models translate between scales, such that the population-level impact of a given individual-level mechanism of interaction can be simulated and predicted. To illustrate the relevance of such models, we formulated two basic models of SARS-CoV-2 interaction (see more details and equations in the Supporting information), with either an endemic colonizing bacterium (parametrized to represent the pneumococcus) or an epidemic respiratory virus (parametrized to represent influenza) circulating prior to SARS-CoV-2. In both cases, we assumed a nonsymmetric (i.e., no effect of SARS-CoV-2 on the other pathogen) interaction that caused a 1- to 5-fold (strength) decrease or increase (sign) of SARS-CoV-2 transmission (mechanism) from coinfected individuals (duration of interaction equal to the infectious period of the other pathogen). Importantly, the within-host processes causing interaction were not explicitly modeled, but their effects were represented by these interaction parameters; other mechanisms of interaction—impact on susceptibility to infection, duration of infection, or disease severity (Fig 1)—would be similarly modeled by parameters representing relative changes in the corresponding epidemiological parameters. As shown in Fig 5A, we find that even a moderately strong (2-fold) interaction with an endemic colonizing bacterium can substantially affect the dynamics of SARS-CoV-2, increasing its peak incidence 1.96-fold for positive interaction when the prevalence of bacterial colonization reaches 50% of the population (as frequently observed in young children for the pneumococcus [97,98]). By contrast, equally strong (2-fold) interaction with an epidemic virus is predicted to have a much smaller maximal impact (1.03-fold increase) on the dynamics of SARS-CoV-2 (Fig 5B). Of note, the maximal impact is predicted at intermediate levels of transmissibility of the epidemic virus, corresponding to maximal epidemic overlap with SARS-CoV-2 (Fig 5B). This finding emphasizes a major difference between endemic and epidemic pathogens: For the latter, the impact of even strong interactions may remain subtle and manifest itself only after a prolonged period of cocirculation with SARS-CoV-2. Overall, these numerical experiments suggest the value of mathematical models to study interactions in a biologically explicit and comprehensive way and to predict their complex (and potentially unexpected) effects at the population level.

Although voluntarily oversimplified and used here only for illustrative and exploratory purposes, these models can be readily extended to add components relevant to SARS-CoV-2 epidemiology, such as age, vaccination, or temporal variations in transmission caused by new variants, seasonality, or changing control measures. In real-world applications, however, model parametrization can be a substantial challenge, as the values of many parameters may be neither directly observable nor fixed from empirical evidence. This problem is particularly salient for parameters characterizing interaction, whose values can be only partially inferred from experimental and epidemiological studies. To overcome this uncertainty, novel statistical inference techniques can be used to systematically compare the likelihood of different hypotheses about the mechanism, strength, and duration of interaction [99,100]. The potential of this approach is demonstrated by earlier successful applications [101,102], in particular to the system influenza–pneumococcus [33,34,103]. So far, however, few modeling studies have attempted to estimate the interactions of SARS-CoV-2 [94,104], presumably because of the near disappearance of many common diseases—caused, for example, by influenza and the pneumococcus [40,41]—after the implementation of stringent control measures against COVID-19. In light of the likely relaxation of these measures and the ensuing increase in cocirculating pathogens, we anticipate that confronting mathematical models with detailed epidemiological surveillance data will increasingly provide valuable insights into the interactions of SARS-CoV-2. Based on the modeling literature cited above and our own experience, we expect such estimation to succeed with routine longitudinal data collected at a fine temporal scale (e.g., weekly counts of infections or hospitalizations), possibly supplemented with other cross-sectional or longitudinal data streams (e.g., seroprevalence or multiplex PCR data to inform the prevalence of prior infections or active coinfections).

## 5. Conclusions

As population immunity against COVID-19 accrues in many regions worldwide, it is critical to understand the factors that will affect the future transmission dynamics of SARS-CoV-2 [2]. Here, we proposed that interactions with cocirculating pathogens will be such a key factor. Indeed, such interactions may have notable public health implications, in particular for forecasting and controlling SARS-CoV-2 epidemics and for predicting the off-target impacts of vaccines. The scientific implications of interaction are also notable and may lead to considering SARS-CoV-2 as part of polymicrobial systems whose individual components cannot be well studied separately.

Despite the relevance of interaction, our review identified only a few experimental studies in animal models, with markedly different designs and the majority focusing on SARS-CoV-2 interaction with IAV. A robust finding from our comparative analysis is that SARS-CoV-2 and IAV coinfections can increase the severity of COVID-19. By contrast, the estimated effect of coinfection on influenza and SARS-CoV-2 viral loads differed markedly across studies, presumably because of the heterogeneous designs and methods to quantify viral load. Perhaps less surprisingly, the design and the results of epidemiological studies on interaction also varied widely. Although previous influenza vaccination was generally associated with a reduced risk of COVID-19, this finding alone does not necessarily provide evidence of positive interaction and may be equally well explained by direct, nonspecific effects of influenza vaccines on host immunity. Nevertheless, the evidence from epidemiological [90] and mathematical modeling [94] studies suggests a facilitatory effect of previous influenza infection on SARS-CoV-2 infection. Besides influenza, few studies investigated the impact of other pathogens, in particular, other major respiratory viruses like RSV and rhinoviruses, or colonizing bacteria like the pneumococcus [105]. In particular, research specific to interactions with endemic bacteria is called for, because—as illustrated by our simple model—these could substantially affect the dynamics of SARS-CoV-2. As a more general conclusion, our review suggests the urgent need for further experimental and epidemiological studies to unequivocally infer SARS-CoV-2 interactions.

Altogether, our review highlights the significant gaps that remain in our knowledge of SARS-CoV-2 interactions. The general framework proposed to dissect interaction may therefore be useful to guide further research in this field. We argue that mathematical models of transmission offer an intrinsically efficient way to incorporate this framework. Hence, we submit that such models—designed with a multidisciplinary perspective that integrates evidence across scientific fields—will prove to be valuable tools to decipher the interactions of SARS-CoV-2.

## Supporting information

S1 Table An overview of the experimental designs and results on disease severity, measured as maximal body mass loss or survival at experiment end, from the reviewed studies assessing the interaction between SARS-CoV-2 and influenza A virus (IAV). (PDF)

S2 Table An overview of the experimental designs and results on viral load, measured in the upper or lower respiratory tract, from the reviewed studies assessing the interaction between SARS-CoV-2 and influenza A virus (IAV). (PDF)

S3 Table Observational studies examining the association between pneumococcal vaccination history and COVID-19. (PDF)

S4 Table Observational studies examining the association between prior respiratory infections and COVID-19. (PDF)

S1 Appendix Model details (including Fig A. Schematic of the bacteria–virus interaction model; Table A. Parameters used for *S*. *pneumoniae*–SARS-CoV-2 interaction model; Fig B. Schematic of the virus–virus interactions model; and Table B. Parameters used for influenza A–SARS-CoV-2 interaction model). (PDF)
