# Supplementary material for: The interactions of SARS-CoV-2 with cocirculating pathogens: Epidemiological implications and current knowledge gaps
Source: PLoS Pathog. 2023 Mar 8;19(3):e1011167. doi: 10.1371/journal.ppat.1011167 (PMC9994710; doi:10.1371/journal.ppat.1011167)
Supplement: S1 Table — (PDF) [file ppat.1011167.s001.pdf]

S1 Table. An overview of the experimental designs and results on disease severity, measured as maximal body mass loss or survival at experiment end, from the reviewed studies assessing the interaction between SARS-CoV-2 and influenza A virus (IAV). Values were taken from tables or text, or when these were not available, extracted from the figures using the program PlotDigitizer [12].

Author	Year	Animal model	Sex	IAV (strain)	SC2 (Pangolin lineage)	SC2 infection order	Days btwn infections	Sample size per group (IAV-SC2-Coinf)	Inoculation dose		Follow-up days	Max body mass loss							Survival at experiment end			
									IAV - SC2 extracted	IAV - SC2 [PFU][13]		IAV	Day IAV	SC2	Day SC2	Coinf	Day Coinf	Figure/Table	IAV	SC2	Coinf	Figure/Table
Bai et al. [1]	2021	K18-hACE2 mice	male	H1N1 (A/Sichuan/01/2009)	SC2 (B)	2nd	2	4-4-4	2X10^3 PFU - 3X10^5 PFU	2X10^3 - 3X10^5	4	NA	NA	-10,0%	4	-10,0%	4	Fig 2B	NA	NA	NA	NA
Bao et al. [2]	2021	Ferrets	male	H1N1 (A/California/07/2009)	SC2 (B)	2nd	5	6-6-6	1X10^6 CCID50 - 1X10^6 CCID50	7X10^5 - 7X10^5	10	-10,0%	6	-0,5%	3	-9,1%	6	Fig1B	NA	NA	NA	NA
Bao et al. [2]	2021	K18-hACE2 mice	female	H1N1 (A/California/07/2009)	SC2 (B)	2nd	5	6-6-6	1X10^3 CCID50 - 1X10^2 CCID50	7X10^2 - 7X10^1	14	-26,0%	9	-24,5%	8	-30,0%	6	Fig 4C	17,0%	0,0%	0,0%	Fig 4B/C
Zhang et al. [3]	2021	Syrian hamster	male + female	H1N1 (A/Hong Kong/415742/2009)	SC2 (B)	simultaneous	0	3-3-3	1X10^5 PFU - 1X10^3 PFU	1X10^5 - 1X10^3	4	-7,0%	2	-8,4%	4	-12,5%	4	Fig 2A	NA	NA	NA	NA
Zhang et al. [3]	2021	Syrian hamster	male + female	H1N1 (A/Hong Kong/415742/2009)	SC2 (B)	simultaneous	0	3-3-3	1X10^4 PFU - 1X10^1 PFU	1X10^4 - 1X10^1	4	4,9%	4	-5,8%	4	-9,6%	4	Fig 3A, Table 1	NA	NA	NA	NA
Zhang et al. [3]	2021	Syrian hamster	male + female	H1N1 (A/Hong Kong/415742/2009)	SC2 (B)	1st	-1	3-3-3	1X10^4 PFU - 1X10^1 PFU	1X10^4 - 1X10^1	4	4,9%	4	-5,8%	4	-11,9%	4	Fig 3A, Table 1	NA	NA	NA	NA
Zhang et al. [3]	2021	Syrian hamster	male + female	H1N1 (A/Hong Kong/415742/2009)	SC2 (B)	2nd	1	3-3-3	1X10^4 PFU - 1X10^1 PFU	1X10^4 - 1X10^1	4	4,9%	4	-5,8%	4	-10,4%	4	Fig 3A, Table 1	NA	NA	NA	NA
Achdout et al. [4]	2021	K18-hACE2 mice	female	H1N1 (A/Puerto Rico/8/1934, PR8)	SC2 (B.1)	2nd	2	10-13-11	8X10^1 PFU - 1X10^1 PFU	8X10^1 - 1X10^1	20-24	-25,0%	10	-5,0%	9	-29,0%	8	Fig 1A	100,0%	62,0%	0,0%	Fig 1B
Achdout et al. [4]	2021	K18-hACE2 mice	female	H1N1 (A/Puerto Rico/8/1934, PR8)	SC2 (B.1)	2nd	5	11-14-7	8X10^1 PFU - 1X10^1 PFU	8X10^1 - 1X10^1	20-24	-21,0%	8	-5,0%	11	-27,0%	10	Fig 1C	100,0%	57,0%	30,0%	Fig 1D
Achdout et al. [4]	2021	K18-hACE2 mice	female	H1N1 (A/Puerto Rico/8/1934, PR8)	SC2 (B.1)	2nd	8	8-14-7	8X10^1 PFU - 1X10^1 PFU	8X10^1 - 1X10^1	20-24	-23,0%	9	-5,0%	14	-25,0%	8	Fig 1E	100,0%	57,0%	57,0%	Fig 1F
Achdout et al. [4]	2021	K18-hACE2 mice	female	H1N1 (A/Puerto Rico/8/1934, PR8)	SC2 (B.1)	2nd	2	4-6-4	8X10^1 PFU - 1X10^1 PFU	8X10^1 - 1X10^1	20-24	-23,0%	9	-5,0%	9	-32,0%	8	Fig 4C	100,0%	50,0%	0,0%	Fig 4B
Achdout et al. [4]	2021	K18-hACE2 mice	female	H1N1 (A/Puerto Rico/8/1934, PR8)	SC2 (B.1)	2nd	2	7-9-9	8X10^1 PFU - 1X10^1 PFU	8X10^1 - 1X10^1	20-24	-28,0%	10	-5,0%	7	-27%*	7	Fig 4F	100,0%	65,0%	10,0%	Fig 4E
Kinoshita et al. [5]	2021	Syrian hamster	female	H1N1 (A/Puerto Rico/8/1934, PR8)	SC2 (B.1.1)	simultaneous	0	6-6-6	1X10^5 PFU - 3X10^5 PFU	1X10^5 - 3X10^5	10	-3,0%	3	-10,0%	6	-14,0%	7	Fig 1A	NA	NA	NA	NA
Li et al. [6]	2021	hACE2 mice	female	H1N1 (A/Puerto Rico/8/1934, PR8)	SC2 (A)	1st	-7	9-9-9	1X10^2 CCID50 - 5X10^3 CCID50	7X10^1 - 3.5X10^3	7	-13,0%	7	NA	NA	-23,0%	7	Fig 3A	NA	NA	NA	NA
Li et al. [6]	2021	hACE2 mice	female	H1N1 (A/Puerto Rico/8/1934, PR8)	SC2 (A)	1st	-14	9-9-9	1X10^2 CCID50 - 5X10^3 CCID50	7X10^1 - 3.5X10^3	14	-13,0%	7	NA	NA	-20,0%	7	Fig 3A	NA	NA	NA	NA
Halfmann et al. [7]	2021	Syrian hamster	female	H3N2 (A/Tokyo/UT-IMS3-1/2014)	SC2 (B.1)	simultaneous	0	NA	1X10^6 PFU - 1X10^3 PFU	1X10^6 - 1X10^3	NA	NA	NA	NA	NA	NA	NA	NA	NA	NA	NA	NA
Halfmann et al. [7]	2021	Syrian hamster	female	H3N2 (A/Tokyo/UT-IMS3-1/2014)	SC2 (B.1)	1st	-10	NA	1X10^6 PFU - 1X10^3 PFU	1X10^6 - 1X10^3	NA	NA	NA	NA	NA	NA	NA	NA	NA	NA	NA	NA
Halfmann et al. [7]	2021	Syrian hamster	female	H3N2 (A/Tokyo/UT-IMS3-1/2014)	SC2 (B.1)	2nd	10	NA	1X10^6 PFU - 1X10^3 PFU	1X10^6 - 1X10^3	NA	NA	NA	NA	NA	NA	NA	NA	NA	NA	NA	NA
Kim et al. [8]	2022	K18-hACE2 mice	female	H1N1 (A/California/04/2009)	SC2 (A)	1st	-3	29-29-26	1X10^4 TCID50 - 1X10^5.5 TCID50	7X10^3 - 7X10^4.5	10	-17,6%	9	-14,7%	7	-27,0%	10	Fig 1B	75,0%	87,5%	0,0%	Fig 1C
Kim et al. [8]	2022	K18-hACE2 mice	female	H1N1 (A/California/04/2009)	SC2 (A)	2nd	3	29-29-26	1X10^4 TCID50 - 1X10^5.5 TCID50	7X10^3 - 7X10^4.5	10	-17,6%	9	-14,7%	7	-21,8%	10	Fig 1B	75,0%	87,5%	0,0%	Fig 1C
Huang et al. [9]	2022	Ferrets	female	H1N1 (A/California/07/2009)	SC2 (A)	simultaneous	0	4	1X10^6 PFU - 5X10^5 PFU	1X10^6 - 5X10^5	14	-12,2%	6	-2,4%	14	-17,3%	7	Fig 1B	NA	NA	NA	NA
Huang et al. [9]	2022	Ferrets	female	H3N2 (A/Kansas/14/2017)	SC2 (A)	simultaneous	0	4	1.3X10^9 PFU - 5X10^5 PFU	1.3X10^9 - 5X10^5	14	-3,1%	5	-2,4%	14	-5,6%	14	Fig 1B	NA	NA	NA	NA
Huang et al. [9]	2022	Ferrets	female	H1N1 (A/California/07/2009)	SC2 (A)	simultaneous	0	4	1X10^6 PFU - 5X10^5 PFU	1X10^6 - 5X10^5	13	-19,7%	7	-1,5%	5	-17,9%	7	Fig 5B/C/F	NA	NA	NA	NA
Huang et al. [9]	2022	Ferrets	female	H3N2 (A/Kansas/14/2017)	SC2 (A)	simultaneous	0	4	1.3X10^9 PFU - 5X10^5 PFU	1.3X10^9 - 5X10^5	13	-3,3%	5	-1,5%	5	-5,4%	14	Fig 5D/E/F	NA	NA	NA	NA
Kim et al. [10]	2022	Syrian hamster	male	H1N1 (A/California/04/2009)	SC2 (B)	1st	-1	Unreported	1X10^5 TCID50 - 1X10^5 TCID50	7X10^4 - 7X10^4	7	-4,2%	1	-12,8%	5	-14,7%	3	Fig 1B	NA	NA	NA	NA
Kim et al. [10]	2022	Syrian hamster	male	H1N1 (A/California/04/2009)	SC2 (B)	2nd	1	Unreported	1X10^5 TCID50 - 1X10^5 TCID50	7X10^4 - 7X10^4	7	-4,2%	1	-12,8%	5	-17,1%	7	Fig 1B	NA	NA	NA	NA
Oishi et al. [11]	2022	Syrian hamster	male	H1N1 (A/California/04/2009)	SC2 (A)	simultaneous	0	8-8-8	1X10^5 PFU - 1x10^3 PFU	1X10^5 - 1x10^3	8	5,2%	3	-2,5%	3	-3,4%	3	Fig 2D	NA	NA	NA	NA
Oishi et al. [11]	2022	Syrian hamster	male	H1N1 (A/California/04/2009)	SC2 (A)	1st	-3	NA	1X10^5 PFU - 1x10^3 PFU	1X10^5 - 1x10^3	NA	NA	NA	NA	NA	NA	NA	NA	NA	NA	NA	NA
Oishi et al. [11]	2022	Syrian hamster	male	H1N1 (A/California/04/2009)	SC2 (A)	2nd	3	NA	1X10^5 PFU - 1x10^3 PFU	1X10^5 - 1x10^3	NA	NA	NA	NA	NA	NA	NA	NA	NA	NA	NA	NA
Oishi et al. [11]	2022	Syrian hamster	male	H1N1 (A/California/04/2009)	SC2 (A)	2nd	7	NA	1X10^5 PFU - 1x10^3 PFU	1X10^5 - 1x10^3	NA	NA	NA	NA	NA	NA	NA	NA	NA	NA	NA	NA
Oishi et al. [11]	2022	Syrian hamster	male	H1N1 (A/California/04/2009)	SC2 (A)	2nd	14	NA	1X10^5 PFU - 1x10^3 PFU	1X10^5 - 1x10^3	NA	NA	NA	NA	NA	NA	NA	NA	NA	NA	NA	NA

Abbreviations K18-hACE2 mice: transgenic mice expressing human angiotensin-converting enzyme 2 (hACE2) controlled by the human cytokeratin 18 promoter, PFU: plaque-forming unit, CCID50: Cell culture infectious dose 50%, TCID50: Tissue culture infectious dose 50%.

***Remark** Viral dose concentrations in PFU following Daelemans et al. [10] protocol (1 CCID50 = 0.7 PFU).

References

1. Bai L, Zhao Y, Dong J, Liang S, Guo M, Liu X, et al. Coinfection with influenza A virus enhances SARS-CoV-2 infectivity. Cell Res. 2021 Apr;31(4):395–403.
2. Bao L, Deng W, Qi F, Lv Q, Song Z, Liu J, et al. Sequential infection with H1N1 and SARS-CoV-2 aggravated COVID-19 pathogenesis in a mammalian model, and co-vaccination as an effective method of prevention of COVID-19 and influenza. Signal Transduct Target Ther. 2021 May 20;6(1):200.
3. Zhang AJ, Lee ACY, Chan JFW, Liu F, Li C, Chen Y, et al. Coinfection by Severe Acute Respiratory Syndrome Coronavirus 2 and Influenza A(H1N1)pdm09 Virus Enhances the Severity of Pneumonia in Golden Syrian Hamsters. Clin Infect Dis. 2021 Jun 15;72(12):e978–92.
4. Achdout H, Vitner EB, Politi B, Melamed S, Yahalom-Ronen Y, Tamir H, et al. Increased lethality in influenza and SARS-CoV-2 coinfection is prevented by influenza immunity but not SARS-CoV-2 immunity. Nat Commun. 2021 Oct 5;12(1):5819.
5. Kinoshita T, Watanabe K, Sakurai Y, Nishi K, Yoshikawa R, Yasuda J. Co-infection of SARS-CoV-2 and influenza virus causes more severe and prolonged pneumonia in hamsters. Sci Rep. 2021 Oct 28;11(1):21259.
6. Li H, Zhao X, Zhao Y, Li J, Zheng H, Xue M, et al. H1N1 exposure during the convalescent stage of SARS-CoV-2 infection results in enhanced lung pathologic damage in hACE2 transgenic mice. Emerg Microbes Infect. 2021 Dec;10(1):1156–68.
7. Halfmann P, Nakajima N, Sato Y, Takahashi K, Accola M, Chibo S, et al. SARS-CoV-2 Interference of Influenza Virus Replication in Syrian Hamsters. J Infect Dis [Internet]. 2021 Dec 7; Available from: http://dx.doi.org/10.1093/infdis/jiab587
8. Kim EH, Nguyen TQ, Casel MAB, Rollon R, Kim SM, Kim YI, et al. Coinfection of SARS-CoV-2 and Influenza A virus increased disease severity, impaired neutralizing antibody, and CD4+ T cell responses. J Virol. 2022 Feb 2;jvi0187321.
9. Huang Y, Skarlupka AL, Jang H, Blas-Machado U, Holladay N, Hogan RJ, et al. SARS-CoV-2 and Influenza A Virus Coinfections in Ferrets. J Virol. 2022 Mar 9;96(5):e0179121.
10. Kim HK, Kang JA, Lyoo KS, Le TB, Yeo YH, Wong SS, et al. Severe acute respiratory syndrome coronavirus 2 and influenza A virus co-infection alters viral tropism and haematological composition in Syrian hamsters. Transbound Emerg Dis [Internet]. 2022 Jun 1; Available from: http://dx.doi.org/10.1111/tbed.14601
11. Oishi K, Horiuchi S, Minkoff JM, tenOever BR. The Host Response to Influenza A Virus Interferes with SARS-CoV-2 Replication during Coinfection. J Virol. 2022 Aug 10;96(15):e0076522.
12. PlotDigitizer: Version 3.1.4. 2022.
13. Daelemans D, Pauwels R, De Clercq E, Pannecouque C. A time-of-drug addition approach to target identification of antiviral compounds. Nat Protoc. 2011 Jun;6(6):925–33.
