# Supplementary material for: The interactions of SARS-CoV-2 with cocirculating pathogens: Epidemiological implications and current knowledge gaps
Source: PLoS Pathog. 2023 Mar 8;19(3):e1011167. doi: 10.1371/journal.ppat.1011167 (PMC9994710; doi:10.1371/journal.ppat.1011167)
Supplement: S2 Table — (PDF) [file ppat.1011167.s002.pdf]

S2 Table. An overview of the experimental designs and results on viral load, measured in the upper or lower respiratory tract, from the reviewed studies assessing the interaction between SARS-CoV-2 and influenza A virus (IAV). Values were obtained from tables or text, or when these were not available, from figures

Author	Year	Animal model	Sex	IAV (strain)	SC2 (Pangolin lineage)	SC2 infection order	Days btwn infections	Sample size (per group)	Inoculation dose	Viral load in lower respiratory tract								Viral load in upper respiratory tract								Quantification Unit	Quantification Method
										Tissue	Sample	Sampling day (dpi)	Coinf (IAV)	Monoinf (IAV)	Coinf (SC2)	Monoinf (SC2)	Figure/ Table	Tissue	Sample	Sampling day (dpi)	Coinf (IAV)	Monoinf (IAV)	Coinf (SC2)	Monoinf (SC2)	Figure/ Table		
Bai et al. [1]	2021	K18-hACE2 mice	male	H1N1 (A/Sichuan/01/2009)	SC2 (B)	2nd	2	3	See table S1	Lung	Tissue	2	NA	NA	6,60	1,00	Fig 2D	NA	NA	NA	NA	NA	NA	NA	NA	gc/GAPDH	RT-qPCR
Bao et al. [2]	2021	Ferrets	male	H1N1 (A/California/07/2009)	SC2 (B)	2nd	5	4	See table S1	Lung	Tissue	5	NA	NA	1,20	3,20	Fig 2C	Throat	Swabs	3	5,80	6,20	5,50	5,40	Fig 2A/B	log10 gc/mL	RT-qPCR
Bao et al. [2]	2021	Ferrets	male	H1N1 (A/California/07/2009)	SC2 (B)	2nd	5	4	See table S1	NA	NA	NA	NA	NA	NA	NA	NA	Throat	Swabs	5	5,40	5,30	4,90	4,90	Fig 2A/B	log10 gc/mL	RT-qPCR
Bao et al. [2]	2021	Ferrets	male	H1N1 (A/California/07/2009)	SC2 (B)	2nd	5	4	See table S1	NA	NA	NA	NA	NA	NA	NA	NA	Throat	Swabs	8	4,80	2,90	0,90	0,00	Fig 2A/B	log10 gc/mL	RT-qPCR
Bao et al. [2]	2021	Ferrets	male	H1N1 (A/California/07/2009)	SC2 (B)	2nd	5	4	See table S1	NA	NA	NA	NA	NA	NA	NA	NA	Throat	Swabs	10	2,00	0,00	0,00	0,00	Fig 2A/B	log10 gc/mL	RT-qPCR
Zhang et al. [3]	2021	Syrian hamster	male + female	H1N1 (A/Hong Kong/415742/2009)	SC2 (B)	simultaneous	0	3	See table S1: High dose	Lung	Tissue	4	4,78	4,86	5,21	5,82	Fig 2C/D	Nasal Turbinates	Tissue	4	4,79	4,00	5,80	4,55	Fig 2C/D	log10 PFU/mL	Plaque-based
Zhang et al. [3]	2021	Syrian hamster	male + female	H1N1 (A/Hong Kong/415742/2009)	SC2 (B)	simultaneous	0	3	See table S1: High dose	Lung	Tissue	4	-1,37	-1,12	0,04	0,45	Fig 2C/D	Nasal Turbinates	Tissue	4	-0,94	0,16	1,56	1,15	Fig 2C/D	log10 gc/beta-actin	RT-qPCR
Zhang et al. [3]	2021	Syrian hamster	male + female	H1N1 (A/Hong Kong/415742/2009)	SC2 (B)	simultaneous	0	3	See table S1: Low dose	Lung	Tissue	4	3,82	2,61	3,55	4,62	Table 1	Nasal Turbinates	Tissue	4	2,24	3,17	5,61	5,48	Table 1	log10 gc/beta-actin	RT-qPCR
Zhang et al. [3]	2021	Syrian hamster	male + female	H1N1 (A/Hong Kong/415742/2009)	SC2 (B)	simultaneous	0	3	See table S1: Low dose	Lung	Tissue	4	3,41	3,98	4,19	5,36	Table 1	Nasal Turbinates	Tissue	4	3,25	3,46	5,14	5,00	Table 1	log10 PFU/mL	Plaque-based
Zhang et al. [3]	2021	Syrian hamster	male + female	H1N1 (A/Hong Kong/415742/2009)	SC2 (B)	1st	-1	3	See table S1: Low dose	Lung	Tissue	4	2,23	2,61	4,71	4,62	Table 1	Nasal Turbinates	Tissue	4	1,56	3,17	5,25	5,48	Table 1	log10 gc/beta-actin	RT-qPCR
Zhang et al. [3]	2021	Syrian hamster	male + female	H1N1 (A/Hong Kong/415742/2009)	SC2 (B)	1st	-1	3	See table S1: Low dose	Lung	Tissue	4	4,36	3,98	5,74	5,36	Table 1	Nasal Turbinates	Tissue	4	3,60	3,46	4,71	5,00	Table 1	log10 PFU/mL	Plaque-based
Zhang et al. [3]	2021	Syrian hamster	male + female	H1N1 (A/Hong Kong/415742/2009)	SC2 (B)	2nd	1	3	See table S1: Low dose	Lung	Tissue	4	4,32	2,61	2,41	4,62	Table 1	Nasal Turbinates	Tissue	4	4,06	3,17	5,45	5,48	Table 1	log10 gc/beta-actin	RT-qPCR
Zhang et al. [3]	2021	Syrian hamster	male + female	H1N1 (A/Hong Kong/415742/2009)	SC2 (B)	2nd	1	3	See table S1: Low dose	Lung	Tissue	4	4,48	3,98	3,65	5,36	Table 1	Nasal Turbinates	Tissue	4	4,43	3,46	5,70	5,00	Table 1	log10 PFU/mL	Plaque-based
Zhang et al. [3]	2021	Syrian hamster	male + female	H1N1 (A/Hong Kong/415742/2009)	SC2 (B)	simultaneous	0	3	See table S1: Low dose	Lung	Tissue	7	0/NA	-3,74	-2,74	-2,27	Fig 4G	Nasal Turbinates	Tissue	7	-4,75	-2,99	0,14	0,41	Fig 4G	log10 gc/beta-actin	RT-qPCR
Zhang et al. [3]	2021	Syrian hamster	male + female	H1N1 (A/Hong Kong/415742/2009)	SC2 (B)	simultaneous	0	3	See table S1: Low dose	Lung	Tissue	14	0/NA	0/NA	0/NA	0/NA	Fig 4G	Nasal Turbinates	Tissue	14	0/NA	0/NA	-2,55	-2,67	Fig 4G	log10 gc/beta-actin	RT-qPCR
Zhang et al. [3]	2021	Syrian hamster	male + female	H1N1 (A/Hong Kong/415742/2009)	SC2 (B)	simultaneous	0	3	See table S1: Low dose	NA	NA	NA	NA	NA	NA	NA	NA	Nasal Turbinates	Swabs	2	3,53	NA	5,38	5,37	Fig 4H	log10 gc/beta-actin	RT-qPCR
Zhang et al. [3]	2021	Syrian hamster	male + female	H1N1 (A/Hong Kong/415742/2009)	SC2 (B)	simultaneous	0	3	See table S1: Low dose	NA	NA	NA	NA	NA	NA	NA	NA	Nasal Turbinates	Swabs	4	2,81	NA	5,38	5,29	Fig 4H	log10 gc/beta-actin	RT-qPCR
Zhang et al. [3]	2021	Syrian hamster	male + female	H1N1 (A/Hong Kong/415742/2009)	SC2 (B)	simultaneous	0	3	See table S1: Low dose	NA	NA	NA	NA	NA	NA	NA	NA	Nasal Turbinates	Swabs	6	3,26	3,02	3,79	3,90	Fig 4H	log10 gc/beta-actin	RT-qPCR
Zhang et al. [3]	2021	Syrian hamster	male + female	H1N1 (A/Hong Kong/415742/2009)	SC2 (B)	simultaneous	0	3	See table S1: Low dose	NA	NA	NA	NA	NA	NA	NA	NA	Nasal Turbinates	Swabs	8	0/NA	0/NA	3,74	4,32	Fig 4H	log10 gc/beta-actin	RT-qPCR
Zhang et al. [3]	2021	Syrian hamster	male + female	H1N1 (A/Hong Kong/415742/2009)	SC2 (B)	simultaneous	0	3	See table S1: Low dose	NA	NA	NA	NA	NA	NA	NA	NA	Nasal Turbinates	Swabs	10	0/NA	0/NA	3,36	0/NA	Fig 4H	log10 gc/beta-actin	RT-qPCR
Zhang et al. [3]	2021	Syrian hamster	male + female	H1N1 (A/Hong Kong/415742/2009)	SC2 (B)	simultaneous	0	3	See table S1: Low dose	NA	NA	NA	NA	NA	NA	NA	NA	Nasal Turbinates	Swabs	12	0/NA	0/NA	0/NA	0/NA	Fig 4H	log10 gc/beta-actin	RT-qPCR
Zhang et al. [3]	2021	Syrian hamster	male + female	H1N1 (A/Hong Kong/415742/2009)	SC2 (B)	simultaneous	0	3	See table S1: Low dose	NA	NA	NA	NA	NA	NA	NA	NA	Nasal Turbinates	Swabs	14	0/NA	0/NA	0/NA	0/NA	Fig 4H	log10 gc/beta-actin	RT-qPCR
Achdout et al. [4]	2021	K18-hACE2 mice	female	H1N1 (A/Puerto Rico/8/1934, PR8)	SC2 (B.1)	2nd	2	10	See table S1	Lung	Tissue	2	4.6**		4,56E+04	6,73E+04	Fig 2B/D	Nasal Turbinates	Tissue	2	11**		4,88E+04	7,26E+04	Fig 2C/E	PFU/organ	Plaque-based
Achdout et al. [4]	2021	K18-hACE2 mice	female	H1N1 (A/Puerto Rico/8/1934, PR8)	SC2 (B.1)	2nd	2	10	See table S1	Lung	Tissue	4	4,19E+08	3,15E+08	7,47E+04	1,50E+04	Fig 2B/D	Nasal Turbinates	Tissue	4	2.3**		1,64E+04	5,52E+04	Fig 2C/E	PFU/organ	Plaque-based
Kinoshita et al. [5]	2021	Syrian hamster	female	H1N1 (A/Puerto Rico/8/1934, PR8)	SC2 (B.1.1)	simultaneous	0	6	See table S1	Lung	Tissue	4	7,00	6,90	11,00	11,00	Table 1	NA	NA	NA	NA	NA	NA	NA	NA	log10 gc/mg	RT-qPCR
Kinoshita et al. [5]	2021	Syrian hamster	female	H1N1 (A/Puerto Rico/8/1934, PR8)	SC2 (B.1.1)	simultaneous	0	6	See table S1	Lung	Tissue	7	3,70	2,70	7,30	7,10	Table 1	NA	NA	NA	NA	NA	NA	NA	NA	log10 gc/mg	RT-qPCR
Li et al. [6]	2021	hACE2 mice	female	H1N1 (A/Puerto Rico/8/1934, PR8)	SC2 (A)	1st	-7	3	See table S1	Lung	Tissue	2	12,66	12,14	NA	NA	Fig 3D	Nasal	Tissue	4	16,58	20,53	NA	NA	Fig 3F	ct	RT-qPCR
Li et al. [6]	2021	hACE2 mice	female	H1N1 (A/Puerto Rico/8/1934, PR8)	SC2 (A)	1st	-7	3	See table S1	Lung	Tissue	4	13,37	13,99	NA	NA	Fig 3D	Nasal	Swabs	1	36,86	37,28	NA	NA	Fig 3B	ct	RT-qPCR
Li et al. [6]	2021	hACE2 mice	female	H1N1 (A/Puerto Rico/8/1934, PR8)	SC2 (A)	1st	-7	3	See table S1	Lung	Tissue	7	17,91	21,60	NA	NA	Fig 3D	Nasal	Swabs	2	29,00	27,68	NA	NA	Fig 3B	ct	RT-qPCR
Li et al. [6]	2021	hACE2 mice	female	H1N1 (A/Puerto Rico/8/1934, PR8)	SC2 (A)	1st	-7	3	See table S1	Trachea	Tissue	2	14,69	14,61	NA	NA	Fig 3E	Nasal	Swabs	3	21,18	23,81	NA	NA	Fig 3B	ct	RT-qPCR
Li et al. [6]	2021	hACE2 mice	female	H1N1 (A/Puerto Rico/8/1934, PR8)	SC2 (A)	1st	-7	3	See table S1	Trachea	Tissue	4	20,16	21,90	NA	NA	Fig 3E	Nasal	Swabs	4	21,70	23,51	NA	NA	Fig 3B	ct	RT-qPCR
Li et al. [6]	2021	hACE2 mice	female	H1N1 (A/Puerto Rico/8/1934, PR8)	SC2 (A)	1st	-7	3	See table S1	Trachea	Tissue	7	23,39	26,73	NA	NA	Fig 3E	Nasal	Swabs	5	20,06	22,18	NA	NA	Fig 3B	ct	RT-qPCR
Li et al. [6]	2021	hACE2 mice	female	H1N1 (A/Puerto Rico/8/1934, PR8)	SC2 (A)	1st	-7	3	See table S1	Pulmonary lymph node	Tissue	4	22,17	26,62	NA	NA	Fig 3F	Nasal	Swabs	6	19,15	20,70	NA	NA	Fig 3B	ct	RT-qPCR
Li et al. [6]	2021	hACE2 mice	female	H1N1 (A/Puerto Rico/8/1934, PR8)	SC2 (A)	1st	-7	3	See table S1	NA	NA	NA	NA	NA	NA	NA	NA	Nasal	Swabs	7	20,33	22,48	NA	NA	Fig 3B	ct	RT-qPCR
Li et al. [6]	2021	hACE2 mice	female	H1N1 (A/Puerto Rico/8/1934, PR8)	SC2 (A)	1st	-7	3	See table S1	NA	NA	NA	NA	NA	NA	NA	NA	Throat	Swabs	1	35,77	35,85	NA	NA	Fig 3C	ct	RT-qPCR
Li et al. [6]	2021	hACE2 mice	female	H1N1 (A/Puerto Rico/8/1934, PR8)	SC2 (A)	1st	-7	3	See table S1	NA	NA	NA	NA	NA	NA	NA	NA	Throat	Swabs	2	33,99	33,37	NA	NA	Fig 3C	ct	RT-qPCR
Li et al. [6]	2021	hACE2 mice	female	H1N1 (A/Puerto Rico/8/1934, PR8)	SC2 (A)	1st	-7	3	See table S1	NA	NA	NA	NA	NA	NA	NA	NA	Throat	Swabs	3	28,74	28,87	NA	NA	Fig 3C	ct	RT-qPCR
Li et al. [6]	2021	hACE2 mice	female	H1N1 (A/Puerto Rico/8/1934, PR8)	SC2 (A)	1st	7	3	See table S1	NA	NA	NA	NA	NA	NA	NA	NA	Throat	Swabs	4	27,06	29,23	NA	NA	Fig 3C	ct	RT-qPCR
Li et al. [6]	2021	hACE2 mice	female	H1N1 (A/Puerto Rico/8/1934, PR8)	SC2 (A)	1st	-7	3	See table S1	NA	NA	NA	NA	NA	NA	NA	NA	Throat	Swabs	5	26,99	28,55	NA	NA	Fig 3C	ct	RT-qPCR
Li et al. [6]	2021	hACE2 mice	female	H1N1 (A/Puerto Rico/8/1934, PR8)	SC2 (A)	1st	-7	3	See table S1	NA	NA	NA	NA	NA	NA	NA	NA	Throat	Swabs	6	28,83	29,79	NA	NA	Fig 3C	ct	RT-qPCR
Li et al. [6]	2021	hACE2 mice	female	H1N1 (A/Puerto Rico/8/1934, PR8)	SC2 (A)	1st	-7	3	See table S1	NA	NA	NA	NA	NA	NA	NA	NA	Throat	Swabs	7	31,28	33,06	NA	NA	Fig 3C	ct	RT-qPCR
Li et al. [6]	2021	hACE2 mice	female	H1N1 (A/Puerto Rico/8/1934, PR8)	SC2 (A)	1st	-14	3	See table S1	Lung	Tissue	2	13,95	12,14	NA	NA	Fig 3D	Nasal	Tissue	4	17,30	20,53	NA	NA	Fig 3F	ct	RT-qPCR
Li et al. [6]	2021	hACE2 mice	female	H1N1 (A/Puerto Rico/8/1934, PR8)	SC2 (A)	1st	-14	3	See table S1	Lung	Tissue	4	15,61	13,99	NA	NA	Fig 3D	Nasal	Swabs	1	34,82	37,28	NA	NA	Fig 3B	ct	RT-qPCR
Li et al. [6]	2021	hACE2 mice	female	H1N1 (A/Puerto Rico/8/1934, PR8)	SC2 (A)	1st	-14	3	See table S1	Lung	Tissue	7	18,55	21,60	NA	NA	Fig 3D	Nasal	Swabs	2	26,41	27,68	NA	NA	Fig 3B	ct	RT-qPCR
Li et al. [6]	2021	hACE2 mice	female	H1N1 (A/Puerto Rico/8/1934, PR8)	SC2 (A)	1st	-14	3	See table S1	Trachea	Tissue	2	14,75	14,61	NA	NA	Fig 3E	Nasal	Swabs	3	23,08	23,81	NA	NA	Fig 3B	ct	RT-qPCR
Li et al. [6]	2021	hACE2 mice	female	H1N1 (A/Puerto Rico/8/1934, PR8)	SC2 (A)	1st	-14	3	See table S1	Trachea	Tissue	4	19,31	21,90	NA	NA	Fig 3E	Nasal	Swabs	4	21,29	23,51	NA	NA	Fig 3B	ct	RT-qPCR
Li et al. [6]	2021	hACE2 mice	female	H1N1 (A/Puerto Rico/8/1934, PR8)	SC2 (A)	1st	-14	3	See table S1	Trachea	Tissue	7	24,31	26,73	NA	NA	Fig 3E	Nasal	Swabs	5	22,10	22,18	NA	NA	Fig 3B	ct	RT-qPCR
Li et al. [6]	2021	hACE2 mice	female	H1N1 (A/Puerto Rico/8/1934, PR8)	SC2 (A)	1st	-14	3	See table S1	Pulmonary lymph node	Tissue	NA	19,92	26,62	NA	NA	Fig 3F	Nasal	Swabs	6	19,25	20,70	NA	NA	Fig 3B	ct	RT-qPCR
Li et al. [6]	2021	hACE2 mice	female	H1N1 (A/Puerto Rico/8/1934, PR8)	SC2 (A)	1st	-14	3	See table S1	NA	NA	NA	NA	NA	NA	NA	NA	Nasal	Swabs	7	24,05	22,48	NA	NA	Fig 3B	ct	RT-qPCR
Li et al. [6]	2021	hACE2 mice	female	H1N1 (A/Puerto Rico/8/1934, PR8)	SC2 (A)	1st	-14	3	See table S1	NA	NA	NA	NA	NA	NA	NA	NA	Throat	Swabs	1	35,42	35,85	NA	NA	Fig 3C	ct	RT-qPCR
Li et al. [6]	2021	hACE2 mice	female	H1N1 (A/Puerto Rico/8/1934, PR8)	SC2 (A)	1st	-14	3	See table S1	NA	NA	NA	NA	NA	NA	NA	NA	Throat	Swabs	2	34,29	33,37	NA	NA	Fig 3C	ct	RT-qPCR
Li et al. [6]	2021	hACE2 mice	female	H1N1 (A/Puerto Rico/8/1934, PR8)	SC2 (A)	1st	-14	3	See table S1	NA	NA	NA	NA	NA	NA	NA	NA	Throat	Swabs	3	28,78	28,87	NA	NA	Fig 3C	ct	RT-qPCR
Li et al. [6]	2021	hACE2 mice	female	H1N1 (A/Puerto Rico/8/1934, PR8)	SC2 (A)	1st	-14	3	See table S1	NA	NA	NA	NA	NA	NA	NA	NA	Throat	Swabs	4	29,01	29,23	NA	NA	Fig 3C	ct	RT-qPCR
Li et al. [6]	2021	hACE2 mice	female	H1N1 (A/Puerto Rico/8/1934, PR8)	SC2 (A)	1st	-14	3	See table S1	NA	NA	NA	NA	NA	NA	NA	NA	Throat	Swabs	5	28,87	28,55	NA	NA	Fig 3C	ct	RT-qPCR
Li et al. [6]	2021	hACE2 mice	female	H1N1 (A/Puerto Rico/8/1934, PR8)	SC2 (A)	1st	-14	3	See table S1	NA	NA	NA	NA	NA	NA	NA	NA	Throat	Swabs	6	31,25	29,79	NA	NA	Fig 3C	ct	RT-qPCR
Li et al. [6]	2021	hACE2 mice	female	H1N1 (A/Puerto Rico/8/1934, PR8)	SC2 (A)	1st	-14	3	See table S1	NA	NA	NA	NA	NA	NA	NA	NA	Throat	Swabs	7	30,52	33,06	NA	NA	Fig 3C	ct	RT-qPCR
Halfmann et al. [7]	2021	Syrian hamster	female	H3N2 (A/Tokyo/UT-IMS3-1/2014)	SC2 (B.1)	simultaneous	0	4	See table S1	Lung	Tissue	3	3,47E+05	7,06E+05	8,73E+07	8,55E+07	Fig 1A	Nasal Turbinates	Tissue	3	1,89E+04	6,57E+05	7,23E+07	7,47E+07	Fig 1A	PFU/g	Plaque-based
Halfmann et al. [7]	2021	Syrian hamster	female	H3N2 (A/Tokyo/UT-IMS3-1/2014)	SC2 (B.1)	1st	-10	4	See table S1	Lung	Tissue	3	2,74E+07	6,09E+07	NA	NA	Fig 1B	Nasal Turbinates	Tissue	3	3,26E+07	7,53E+07	NA	NA	Fig 1B	PFU/g	Plaque-based
Halfmann et al. [7]	2021	Syrian hamster	female	H3N2 (A/Tokyo/UT-IMS3-1/2014)	SC2 (B.1)	1st	-21	4	See table S1	Lung	Tissue	3	3,40E+07	7,21E+07	NA	NA	Fig 1C	Nasal Turbinates	Tissue	3	4,79E+07	7,26E+07	NA	NA	Fig 1C	PFU/g	Plaque-based
Halfmann et al. [7]	2021	Syrian hamster	female	H3N2 (A/Tokyo/UT-IMS3-1/2014)	SC2 (B.1)	2nd	10	4	See table S1	Lung	Tissue	3	NA	NA	7,11E+07	6,72E+07	Fig 1D	Nasal Turbinates	Tissue	3	NA	NA	5,05E+07	4,36E+07	Fig 1D	PFU/g	Plaque-based
Kim et al. [8]	2022	K18-hACE2 mice	female	H1N1 (A/California/04/2009)	SC2 (A)	1st	-3	3	See table S1	Lung	Tissue	2	3,73	5,94	4,47	4,66	Fig 2B/C	NA	NA	NA	NA	NA	NA	NA	NA	log10 TCID50/g	Plaque-based
Kim et al. [8]	2022	K18-hACE2 mice	female	H1N1 (A/California/04/2009)	SC2 (A)	1st	-3	3	See table S1	Lung	Tissue	4	3,37	5,66	4,75	1,52	Fig 2B/C	NA	NA	NA	NA	NA	NA	NA	NA	log10 TCID50/g	Plaque-based
Kim et al. [8]	2022	K18-hACE2 mice	female	H1N1 (A/California/04/2009)	SC2 (A)	1st	-3	3	See table S1	Lung	Tissue	7	3,72	1,52	1,52	1,52	Fig 2B/C	NA	NA	NA	NA	NA	NA	NA	NA	log10 TCID50/g	Plaque-based
Kim et al. [8]	2022	K18-hACE2 mice	female	H1N1 (A/California/04/2009)	SC2 (A)	2nd	3	3	See table S1	Lung	Tissue	2	4,39	5,92	3,71	4,65	Fig 2A/D	NA	NA	NA	NA	NA	NA	NA	NA	log10 TCID50/g	Plaque-based
Kim et al. [8]	2022	K18-hACE2 mice	female	H1N1 (A/California/04/2009)	SC2 (A)	2nd	3	3	See table S1	Lung	Tissue	4	3,09	5,63	2,15	1,52	Fig 2A/D	NA	NA	NA	NA	NA	NA	NA	NA	log10 TCID50/g	Plaque-based
Kim et al. [8]	2022	K18-hACE2 mice	female	H1N1 (A/California/04/2009)	SC2 (A)	2nd	3	3	See table S1	Lung	Tissue	7	2,78	1,52	1,52	1,52	Fig 2A/D	NA	NA	NA	NA	NA	NA	NA	NA	log10 TCID50/g	Plaque-based

Author	Year	Animal model	Sex	IAV (strain)	SC2 (Pangolin lineage)	SC2 infection order	Days btwn infections	Sample size (per group)	Inoculation dose	Viral load in lower respiratory tract								Viral load in upper respiratory tract								Quantification Unit	Quantification Method
										Tissue	Sample	Sampling day (dpi)	Coinf (IAV)	Monoinf (IAV)	Coinf (SC2)	Monoinf (SC2)	Figure/ Table	Tissue	Sample	Sampling day (dpi)	Coinf (IAV)	Monoinf (IAV)	Coinf (SC2)	Monoinf (SC2)	Figure/ Table		
Huang et al. [9]	2022	Ferrets	female	H1N1 (A/California/07/2009)	SC2 (A)	simultaneous	0	4	See table S1	Lung	Tissue	4	4,80	5,35	NA	NA	Figure 3A	Nasal	Swabs	1	3,20	3,89	NA	NA	Fig 2A-D	log10 PFU/mL	Plaque-based
Huang et al. [9]	2022	Ferrets	female	H1N1 (A/California/07/2009)	SC2 (A)	simultaneous	0	4	See table S1	NA	NA	NA	NA	NA	NA	NA	NA	Nasal	Swabs	3	3,06	4,42	NA	NA	Fig 2A-D	log10 PFU/mL	Plaque-based
Huang et al. [9]	2022	Ferrets	female	H1N1 (A/California/07/2009)	SC2 (A)	simultaneous	0	4	See table S1	NA	NA	NA	NA	NA	NA	NA	NA	Nasal	Swabs	5	3,86	4,25	NA	NA	Fig 2A-D	log10 PFU/mL	Plaque-based
Huang et al. [9]	2022	Ferrets	female	H1N1 (A/California/07/2009)	SC2 (A)	simultaneous	0	4	See table S1	NA	NA	NA	NA	NA	NA	NA	NA	Nasal	Swabs	7	1,00	1,00	NA	NA	Fig 2A-D	log10 PFU/mL	Plaque-based
Huang et al. [9]	2022	Ferrets	female	H1N1 (A/California/07/2009)	SC2 (A)	simultaneous	0	4	See table S1	NA	NA	NA	NA	NA	NA	NA	NA	Nasal	Swabs	9	1,00	1,00	NA	NA	Fig 2A-D	log10 PFU/mL	Plaque-based
Huang et al. [9]	2022	Ferrets	female	H3N2 (A/Kansas/14/2017)	SC2 (A)	simultaneous	0	4	See table S1	Lung	Tissue	4	1,00	1,00	NA	NA	Figure 3A	Nasal	Swabs	1	2,29	3,28	NA	NA	Fig 2A-D	log10 PFU/mL	Plaque-based
Huang et al. [9]	2022	Ferrets	female	H3N2 (A/Kansas/14/2017)	SC2 (A)	simultaneous	0	4	See table S1	NA	NA	NA	NA	NA	NA	NA	NA	Nasal	Swabs	3	2,82	3,16	NA	NA	Fig 2A-D	log10 PFU/mL	Plaque-based
Huang et al. [9]	2022	Ferrets	female	H3N2 (A/Kansas/14/2017)	SC2 (A)	simultaneous	0	4	See table S1	NA	NA	NA	NA	NA	NA	NA	NA	Nasal	Swabs	5	1,93	2,32	NA	NA	Fig 2A-D	log10 PFU/mL	Plaque-based
Huang et al. [9]	2022	Ferrets	female	H3N2 (A/Kansas/14/2017)	SC2 (A)	simultaneous	0	4	See table S1	NA	NA	NA	NA	NA	NA	NA	NA	Nasal	Swabs	7	1,00	1,00	NA	NA	Fig 2A-D	log10 PFU/mL	Plaque-based
Huang et al. [9]	2022	Ferrets	female	H3N2 (A/Kansas/14/2017)	SC2 (A)	simultaneous	0	4	See table S1	NA	NA	NA	NA	NA	NA	NA	NA	Nasal	Swabs	9	1,00	1,00	NA	NA	Fig 2A-D	log10 PFU/mL	Plaque-based
Huang et al. [9]	2022	Ferrets	female	H1N1 (A/California/07/2009)	SC2 (A)	simultaneous	0	4	See table S1	Lung	Tissue	4	NA	NA	4,30	4,30	Figure 3B	Nasal	Swabs	1	NA	NA	6,50	5,25	Fig 2E-G	log2 TCID50/mL	Median tissue culture infectious dose
Huang et al. [9]	2022	Ferrets	female	H1N1 (A/California/07/2009)	SC2 (A)	simultaneous	0	4	See table S1	NA	NA	NA	NA	NA	NA	NA	NA	Nasal	Swabs	3	NA	NA	4,30	6,97	Fig 2E-G	log2 TCID50/mL	Median tissue culture infectious dose
Huang et al. [9]	2022	Ferrets	female	H1N1 (A/California/07/2009)	SC2 (A)	simultaneous	0	4	See table S1	NA	NA	NA	NA	NA	NA	NA	NA	Nasal	Swabs	5	NA	NA	4,30	5,87	Fig 2E-G	log2 TCID50/mL	Median tissue culture infectious dose
Huang et al. [9]	2022	Ferrets	female	H1N1 (A/California/07/2009)	SC2 (A)	simultaneous	0	4	See table S1	NA	NA	NA	NA	NA	NA	NA	NA	Nasal	Swabs	7	NA	NA	4,30	4,56	Fig 2E-G	log2 TCID50/mL	Median tissue culture infectious dose
Huang et al. [9]	2022	Ferrets	female	H1N1 (A/California/07/2009)	SC2 (A)	simultaneous	0	4	See table S1	NA	NA	NA	NA	NA	NA	NA	NA	Nasal	Swabs	9	NA	NA	4,30	4,55	Fig 2E-G	log2 TCID50/mL	Median tissue culture infectious dose
Huang et al. [9]	2022	Ferrets	female	H3N2 (A/Kansas/14/2017)	SC2 (A)	simultaneous	0	4	See table S1	Lung	Tissue	4	NA	NA	4,30	4,30	Figure 3B	Nasal	Swabs	1	NA	NA	6,88	5,25	Fig 2E-G	log2 TCID50/mL	Median tissue culture infectious dose
Huang et al. [9]	2022	Ferrets	female	H3N2 (A/Kansas/14/2017)	SC2 (A)	simultaneous	0	4	See table S1	NA	NA	NA	NA	NA	NA	NA	NA	Nasal	Swabs	3	NA	NA	4,30	6,97	Fig 2E-G	log2 TCID50/mL	Median tissue culture infectious dose
Huang et al. [9]	2022	Ferrets	female	H3N2 (A/Kansas/14/2017)	SC2 (A)	simultaneous	0	4	See table S1	NA	NA	NA	NA	NA	NA	NA	NA	Nasal	Swabs	5	NA	NA	4,30	5,87	Fig 2E-G	log2 TCID50/mL	Median tissue culture infectious dose
Huang et al. [9]	2022	Ferrets	female	H3N2 (A/Kansas/14/2017)	SC2 (A)	simultaneous	0	4	See table S1	NA	NA	NA	NA	NA	NA	NA	NA	Nasal	Swabs	7	NA	NA	4,30	4,56	Fig 2E-G	log2 TCID50/mL	Median tissue culture infectious dose
Huang et al. [9]	2022	Ferrets	female	H3N2 (A/Kansas/14/2017)	SC2 (A)	simultaneous	0	4	See table S1	NA	NA	NA	NA	NA	NA	NA	NA	Nasal	Swabs	9	NA	NA	4,30	4,55	Fig 2E-G	log2 TCID50/mL	Median tissue culture infectious dose
Huang et al. [9]	2022	Ferrets	female	H1N1 (A/California/07/2009)	SC2 (A)	simultaneous	0	4	See table S1	NA	NA	NA	NA	NA	NA	NA	NA	Nasal	Swabs	1	NA	NA	3,30	3,16	Fig 8A/C	log10 gc/uL	RT-qPCR
Huang et al. [9]	2022	Ferrets	female	H1N1 (A/California/07/2009)	SC2 (A)	simultaneous	0	4	See table S1	NA	NA	NA	NA	NA	NA	NA	NA	Nasal	Swabs	3	NA	NA	2,18	4,64	Fig 8A/C	log10 gc/uL	RT-qPCR
Huang et al. [9]	2022	Ferrets	female	H1N1 (A/California/07/2009)	SC2 (A)	simultaneous	0	4	See table S1	NA	NA	NA	NA	NA	NA	NA	NA	Nasal	Swabs	5	NA	NA	1,33	3,74	Fig 8A/C	log10 gc/uL	RT-qPCR
Huang et al. [9]	2022	Ferrets	female	H1N1 (A/California/07/2009)	SC2 (A)	simultaneous	0	4	See table S1	NA	NA	NA	NA	NA	NA	NA	NA	Nasal	Swabs	7	NA	NA	1,51	3,40	Fig 8A/C	log10 gc/uL	RT-qPCR
Huang et al. [9]	2022	Ferrets	female	H1N1 (A/California/07/2009)	SC2 (A)	simultaneous	0	4	See table S1	NA	NA	NA	NA	NA	NA	NA	NA	Nasal	Swabs	9	NA	NA	0,48	0,00	Fig 8A/C	log10 gc/uL	RT-qPCR
Huang et al. [9]	2022	Ferrets	female	H3N2 (A/Kansas/14/2017)	SC2 (A)	simultaneous	0	4	See table S1	NA	NA	NA	NA	NA	NA	NA	NA	Nasal	Swabs	1	NA	NA	3,16	3,16	Fig 8B/C	log10 gc/uL	RT-qPCR
Huang et al. [9]	2022	Ferrets	female	H3N2 (A/Kansas/14/2017)	SC2 (A)	simultaneous	0	4	See table S1	NA	NA	NA	NA	NA	NA	NA	NA	Nasal	Swabs	3	NA	NA	2,19	4,64	Fig 8B/C	log10 gc/uL	RT-qPCR
Huang et al. [9]	2022	Ferrets	female	H3N2 (A/Kansas/14/2017)	SC2 (A)	simultaneous	0	4	See table S1	NA	NA	NA	NA	NA	NA	NA	NA	Nasal	Swabs	5	NA	NA	0,54	3,74	Fig 8B/C	log10 gc/uL	RT-qPCR
Huang et al. [9]	2022	Ferrets	female	H3N2 (A/Kansas/14/2017)	SC2 (A)	simultaneous	0	4	See table S1	NA	NA	NA	NA	NA	NA	NA	NA	Nasal	Swabs	7	NA	NA	0,00	3,40	Fig 8B/C	log10 gc/uL	RT-qPCR
Huang et al. [9]	2022	Ferrets	female	H3N2 (A/Kansas/14/2017)	SC2 (A)	simultaneous	0	4	See table S1	NA	NA	NA	NA	NA	NA	NA	NA	Nasal	Swabs	9	NA	NA	0,00	0,00	Fig 8B/C	log10 gc/uL	RT-qPCR
Kim et al. [10]	2022	Syrian hamster	male	H1N1 (A/California/04/2009)	SC2 (B)	1st	-1	Unreported	See table S1	Lung	Tissue	1	1,93	1,88	5,48	4,15	Fig 1E/H	Nasal Turbinates	Tissue	1	1,91	2,99	5,70	5,80	Fig 1C/F	log10 TCID50/mL	RT-qPCR
Kim et al. [10]	2022	Syrian hamster	male	H1N1 (A/California/04/2009)	SC2 (B)	1st	-1	Unreported	See table S1	Lung	Tissue	3	2,68	1,00	4,68	3,93	Fig 1E/H	Nasal Turbinates	Tissue	3	2,42	3,66	4,55	4,61	Fig 1C/F	log10 TCID50/mL	RT-qPCR
Kim et al. [10]	2022	Syrian hamster	male	H1N1 (A/California/04/2009)	SC2 (B)	1st	-1	Unreported	See table S1	Lung	Tissue	6	1,00	1,00	2,81	2,43	Fig 1E/H	Nasal Turbinates	Tissue	6	3,64	1,00	3,62	4,00	Fig 1C/F	log10 TCID50/mL	RT-qPCR
Kim et al. [10]	2022	Syrian hamster	male	H1N1 (A/California/04/2009)	SC2 (B)	1st	-1	Unreported	See table S1	Trachea	Tissue	1	1,93	1,88	3,23	2,63	Fig 1D/G	NA	NA	NA	NA	NA	NA	NA	NA	log10 TCID50/mL	RT-qPCR
Kim et al. [10]	2022	Syrian hamster	male	H1N1 (A/California/04/2009)	SC2 (B)	1st	-1	Unreported	See table S1	Trachea	Tissue	3	2,50	1,00	3,86	1,99	Fig 1D/G	NA	NA	NA	NA	NA	NA	NA	NA	log10 TCID50/mL	RT-qPCR
Kim et al. [10]	2022	Syrian hamster	male	H1N1 (A/California/04/2009)	SC2 (B)	1st	-1	Unreported	See table S1	Trachea	Tissue	6	1,00	1,00	1,73	1,00	Fig 1D/G	NA	NA	NA	NA	NA	NA	NA	NA	log10 TCID50/mL	RT-qPCR
Kim et al. [10]	2022	Syrian hamster	male	H1N1 (A/California/04/2009)	SC2 (B)	2nd	1	Unreported	See table S1	Lung	Tissue	1	1,27	1,88	4,42	4,15	Fig 1E/H	Nasal Turbinates	Tissue	1	2,52	2,99	5,82	5,80	Fig 1C/F	log10 TCID50/mL	RT-qPCR
Kim et al. [10]	2022	Syrian hamster	male	H1N1 (A/California/04/2009)	SC2 (B)	2nd	1	Unreported	See table S1	Lung	Tissue	3	2,32	1,00	4,34	3,93	Fig 1E/H	Nasal Turbinates	Tissue	3	2,17	3,66	4,39	4,61	Fig 1C/F	log10 TCID50/mL	RT-qPCR
Kim et al. [10]	2022	Syrian hamster	male	H1N1 (A/California/04/2009)	SC2 (B)	2nd	1	Unreported	See table S1	Lung	Tissue	6	1,00	1,00	2,39	2,43	Fig 1E/H	Nasal Turbinates	Tissue	6	1,98	1,00	3,65	4,00	Fig 1C/F	log10 TCID50/mL	RT-qPCR
Kim et al. [10]	2022	Syrian hamster	male	H1N1 (A/California/04/2009)	SC2 (B)	2nd	1	Unreported	See table S1	Trachea	Tissue	1	1,30	1,88	1,00	2,63	Fig 1D/G	NA	NA	NA	NA	NA	NA	NA	NA	log10 TCID50/mL	RT-qPCR
Kim et al. [10]	2022	Syrian hamster	male	H1N1 (A/California/04/2009)	SC2 (B)	2nd	1	Unreported	See table S1	Trachea	Tissue	3	2,32	1,00	1,00	1,99	Fig 1D/G	NA	NA	NA	NA	NA	NA	NA	NA	log10 TCID50/mL	RT-qPCR
Kim et al. [10]	2022	Syrian hamster	male	H1N1 (A/California/04/2009)	SC2 (B)	2nd	1	Unreported	See table S1	Trachea	Tissue	6	1,00	1,00	1,00	1,00	Fig 1D/G	NA	NA	NA	NA	NA	NA	NA	NA	log10 TCID50/mL	RT-qPCR
Oishi et al. [11]	2022	Syrian hamster	male	H1N1 (A/California/04/2009)	SC2 (A)	simultaneous	0	4	See table S1	Lung	Tissue	1	5,74	5,66	7,17	7,17	Fig 2B/C	NA	NA	NA	NA	NA	NA	NA	NA	log10 PFU/g	Plaque-based
Oishi et al. [11]	2022	Syrian hamster	male	H1N1 (A/California/04/2009)	SC2 (A)	simultaneous	0	4	See table S1	Lung	Tissue	3	6,90	7,04	6,56	8,07	Fig 2B/C	NA	NA	NA	NA	NA	NA	NA	NA	log10 PFU/g	Plaque-based
Oishi et al. [11]	2022	Syrian hamster	male	H1N1 (A/California/04/2009)	SC2 (A)	simultaneous	0	4	See table S1	Lung	Tissue	5	3,32	3,32	3,12	6,92	Fig 2B/C	NA	NA	NA	NA	NA	NA	NA	NA	log10 PFU/g	Plaque-based
Oishi et al. [11]	2022	Syrian hamster	male	H1N1 (A/California/04/2009)	SC2 (A)	simultaneous	0	4	See table S1	Lung	Tissue	7	3,00	3,11	3,00	3,12	Fig 2B/C	NA	NA	NA	NA	NA	NA	NA	NA	log10 PFU/g	Plaque-based
Oishi et al. [11]	2022	Syrian hamster	male	H1N1 (A/California/04/2009)	SC2 (A)	simultaneous	0	4	See table S1	Lung	Tissue	14	3,00	3,00	3,00	3,00	Fig 2B/C	NA	NA	NA	NA	NA	NA	NA	NA	log10 PFU/g	Plaque-based
Oishi et al. [11]	2022	Syrian hamster	male	H1N1 (A/California/04/2009)	SC2 (A)	1st	-3	3	See table S1	Lung	Tissue	1	4,85	5,49	7,39	7,37	Fig 3E/F	NA	NA	NA	NA	NA	NA	NA	NA	log10 PFU/g	Plaque-based
Oishi et al. [11]	2022	Syrian hamster	male	H1N1 (A/California/04/2009)	SC2 (A)	1st	-3	3	See table S1	Lung	Tissue	3	5,92	6,59	3,12	3,11	Fig 3E/F	NA	NA	NA	NA	NA	NA	NA	NA	log10 PFU/g	Plaque-based
Oishi et al. [11]	2022	Syrian hamster	male	H1N1 (A/California/04/2009)	SC2 (A)	1st	-3	3	See table S1	Lung	Tissue	5	3,92	3,88	3,00	3,00	Fig 3E/F	NA	NA	NA	NA	NA	NA	NA	NA	log10 PFU/g	Plaque-based
Oishi et al. [11]	2022	Syrian hamster	male	H1N1 (A/California/04/2009)	SC2 (A)	2nd	3	3	See table S1	Lung	Tissue	1	7,70	7,24	3,23	7,08	Fig 3B/C	NA	NA	NA	NA	NA	NA	NA	NA	log10 PFU/g	Plaque-based
Oishi et al. [11]	2022	Syrian hamster	male	H1N1 (A/California/04/2009)	SC2 (A)	2nd	3	3	See table S1	Lung	Tissue	3	3,00	3,00	7,49	9,09	Fig 3B/C	NA	NA	NA	NA	NA	NA	NA	NA	log10 PFU/g	Plaque-based
Oishi et al. [11]	2022	Syrian hamster	male	H1N1 (A/California/04/2009)	SC2 (A)	2nd	3	3	See table S1	Lung	Tissue	5	3,00	3,00	3,39	7,31	Fig 3B/C	NA	NA	NA	NA	NA	NA	NA	NA	log10 PFU/g	Plaque-based
Oishi et al. [11]	2022	Syrian hamster	male	H1N1 (A/California/04/2009)	SC2 (A)	2nd	7	4	See table S1	Lung	Tissue	1	3,00	3,00	3,00	6,79	Fig 4B	NA	NA	NA	NA	NA	NA	NA	NA	log10 PFU/g	Plaque-based
Oishi et al. [11]	2022	Syrian hamster	male	H1N1 (A/California/04/2009)	SC2 (A)	2nd	7	4	See table S1	Lung	Tissue	3	3,00	3,00	7,69	8,93	Fig 4B	NA	NA	NA	NA	NA	NA	NA	NA	log10 PFU/g	Plaque-based
Oishi et al. [11]	2022	Syrian hamster	male	H1N1 (A/California/04/2009)	SC2 (A)	2nd	7	4	See table S1	Lung	Tissue	5	3,00	3,00	7,15	7,44	Fig 4B	NA	NA	NA	NA	NA	NA	NA	NA	log10 PFU/g	Plaque-based
Oishi et al. [11]	2022	Syrian hamster	male	H1N1 (A/California/04/2009)	SC2 (A)	2nd	7	4	See table S1	Lung	Tissue	7	3,00	3,00	3,00	3,00	Fig 4B	NA	NA	NA	NA	NA	NA	NA	NA	log10 PFU/g	Plaque-based
Oishi et al. [11]	2022	Syrian hamster	male	H1N1 (A/California/04/2009)	SC2 (A)	2nd	14	4	See table S1	Lung	Tissue	1	3,00	3,00	6,76	8,02	Fig 4D	NA	NA	NA	NA	NA	NA	NA	NA	log10 PFU/g	Plaque-based
Oishi et al. [11]	2022	Syrian hamster	male	H1N1 (A/California/04/2009)	SC2 (A)	2nd	14	4	See table S1	Lung	Tissue	3	3,00	3,00	8,15	8,61	Fig 4D	NA	NA	NA	NA	NA	NA	NA	NA	log10 PFU/g	Plaque-based
Oishi et al. [11]	2022	Syrian hamster	male	H1N1 (A/California/04/2009)	SC2 (A)	2nd	14	4	See table S1	Lung	Tissue	5	3,00	3,00	7,03	7,55	Fig 4D	NA	NA	NA	NA	NA	NA	NA	NA	log10 PFU/g	Plaque-based
Oishi et al. [11]	2022	Syrian hamster	male	H1N1 (A/California/04/2009)	SC2 (A)	2nd	14	4	See table S1	Lung	Tissue	7	3,00	3,00	3,00	3,00	Fig 4D	NA	NA	NA	NA	NA	NA	NA	NA	log10 PFU/g	Plaque-based

Abbreviations K18-hACE2 mice: transgenic mice expressing human angiotensin-converting enzyme 2 (hACE2) controlled by the human cytokeratin 18 promoter, dpi: days post infection, PFU: plaque-forming unit, CCID50: Cell culture infectious dose 50%, TCID50: Tissue culture infectious dose 50%, ct: cycle threshold, gc: gene copies, GAPDH: glyceraldehyde 3-phosphate dehydrogenase, RT-qPCR: reverse transcription quantitative

****Remark** Viral load ratio (co-infection:mono-infection) reported directly in the articles.

References

1. Bai L, Zhao Y, Dong J, Liang S, Guo M, Liu X, et al. Coinfection with influenza A virus enhances SARS-CoV-2 infectivity. Cell Res. 2021 Apr;31(4):395–403.
2. Bao L, Deng W, Qi F, Lv Q, Song Z, Liu J, et al. Sequential infection with H1N1 and SARS-CoV-2 aggravated COVID-19 pathogenesis in a mammalian model, and co-vaccination as an effective method of prevention of COVID-19 and influenza. Signal Transduct Target Ther. 2021 May 20;6(1):200.
3. Zhang AJ, Lee ACY, Chan JFW, Liu F, Li C, Chen Y, et al. Coinfection by Severe Acute Respiratory Syndrome Coronavirus 2 and Influenza A(H1N1)pdm09 Virus Enhances the Severity of Pneumonia in Golden Syrian Hamsters. Clin Infect Dis. 2021 Jun 15;72(12):e978–92.
4. Achdout H, Vitner EB, Politi B, Melamed S, Yahalom-Ronen Y, Tamir H, et al. Increased lethality in influenza and SARS-CoV-2 coinfection is prevented by influenza immunity but not SARS-CoV-2 immunity. Nat Commun. 2021 Oct 5;12(1):5819.
5. Kinoshita T, Watanabe K, Sakurai Y, Nishi K, Yoshikawa R, Yasuda J. Co-infection of SARS-CoV-2 and influenza virus causes more severe and prolonged pneumonia in hamsters. Sci Rep. 2021 Oct 28;11(1):21259.
6. Li H, Zhao X, Zhao Y, Li J, Zheng H, Xue M, et al. H1N1 exposure during the convalescent stage of SARS-CoV-2 infection results in enhanced lung pathologic damage in hACE2 transgenic mice. Emerg Microbes Infect. 2021 Dec;10(1):1156–68.
7. Halfmann P, Nakajima N, Sato Y, Takahashi K, Accola M, Chibo S, et al. SARS-CoV-2 Interference of Influenza Virus Replication in Syrian Hamsters. J Infect Dis [Internet]. 2021 Dec 7; Available from: http://dx.doi.org/10.1093/infdis/jiab587
8. Kim EH, Nguyen TQ, Casel MAB, Rollon R, Kim SM, Kim YI, et al. Coinfection of SARS-CoV-2 and Influenza A virus increased disease severity, impaired neutralizing antibody, and CD4+ T cell responses. J Virol. 2022 Feb 2;jvi0187321.
9. Huang Y, Skarlupka AL, Jang H, Blas-Machado U, Holladay N, Hogan RJ, et al. SARS-CoV-2 and Influenza A Virus Coinfections in Ferrets. J Virol. 2022 Mar 9;96(5):e0179121.
10. Kim HK, Kang JA, Lyoo KS, Le TB, Yeo YH, Wong SS, et al. Severe acute respiratory syndrome coronavirus 2 and influenza A virus co-infection alters viral tropism and haematological composition in Syrian hamsters. Transbound Emerg Dis [Internet]. 2022 Jun 1; Available from: http://dx.doi.org/10.1111/tbed.14601
11. Oishi K, Horiuchi S, Minkoff JM, tenOever BR. The Host Response to Influenza A Virus Interferes with SARS-CoV-2 Replication during Coinfection. J Virol. 2022 Aug 10;96(15):e0076522.
12. PlotDigitizer: Version 3.1.4. 2022.
