# Supplementary material for: The interactions of SARS-CoV-2 with cocirculating pathogens: Epidemiological implications and current knowledge gaps
Source: PLoS Pathog. 2023 Mar 8;19(3):e1011167. doi: 10.1371/journal.ppat.1011167 (PMC9994710; doi:10.1371/journal.ppat.1011167)
Supplement: S3 Table — (PDF) [file ppat.1011167.s003.pdf]

S3 Table. Observational studies examining the association between pneumococcal vaccination history and COVID-19 [1–4].

Author	*Design	Study population	Method to control for confounding	Outcome	Exposure	**Effect Estimate (95% CI)
Lewnard et al. [1]	Cohort-Prospective	Adults ≥65yrs who had been members in a local healthcare system for ≥12mo	Using doubly robust inverse propensity weighting in Cox model, corrected for negative control (zoster vaccine recipients)	COVID-19 diagnosis	PCV<br>PPSV	HR=0.65 (0.59, 0.72)<br>HR=1.19 (1.05, 1.36)
				COVID-19 hospitalization		HR=0.68 (0.57, 0.83)<br>HR=1.02 (0.78, 1.29)
				COVID-19 mortality		HR=0.68 (0.49, 0.95)<br>HR=1.28 (0.77, 2.01)
Patwardhan et al. [2]	Cohort-Retrospective	Children ≤20yrs who were PCR+ for SARS-CoV-2	Adjusted for race, sex, age, month of diagnosis, comorbidity, allergy/asthma, obesity, smoke exposure	Subjective and objective symptom	Flu vac<br>PCV	OR=0.714 (0.529, 0.964)<br>OR=0.482(0.277,0.837)
				Respiratory symptom		OR=0.678(0.492,0.934)<br>OR=0.765(0.428,1.368)
				Severity (objective symptom– vs. objective symptom+)		OR=0.672(0.500, 0.903)<br>OR=0.412(0.234, 0.725)
Rivas et al. [3]	Cohort-Retrospective	HCW who had provided blood sample and with known vaccination status	Adjusted for sex, age	***Blood test: anti–SARS-CoV-2 IgG+ index value >0.4	BCG vac<br>Men vac<br>PPSV<br>Flu vac	OR=0.76 (0.57, 0.99)<br>OR=0.90 (0.69, 1.17)<br>OR=0.99 (0.71, 1.36)<br>OR=1.84 (0.57, 11.27)

Author	Design	Study population	Method to control for confounding	Outcome	Exposure	**Effect Estimate (95% CI)
Fernández-Prada et al. [4]	Case-control	Suspected cases (≥1 epi criterion + ≥1 clinical criterion)	Controls matched to cases based on sex, age, severity (hospital/home)	PCR+ for SARS-CoV-2	Flu vac<br>PCV<br>PPSV	OR=1.7 (0.957-3.254)<br>OR=0.4 (0.170-1.006)<br>OR=0.7 (0.284-2.097)

Abbreviations PCV: pneumococcal conjugate vaccine, PPSV: pneumococcal polysaccharide vaccine, Flu vac: inluenza vaccine, BCG vac: Bacillus Calmette–Guérin vaccine, Men vac: Meningococcal vaccine, HCW: health care workers, PCR: Polymerase chain reaction, CI: confidence intervals, HR: hazard ratio, OR: odds ratio.

***Remark 1** Study design was determined to be cohort if participants were selected based on exposure (study compared outcome in exposed vs. unexposed participants) and case-control if participants were selected based on outcome (study compared exposure in participants with vs. without outcome); cohort-prospective means the information on exposure was recorded before the outcome occurred in the study and cohort-retrospective means the information on exposure was recorded after the outcome occurred.

****Remark 2** Effect estimates were HR or OR directly extracted from studies.

*****Remark 3** The presence of anti–SARS-CoV-2 IgG+ was interpreted as prior SARS-CoV-2 infection in the context before large-scale COVID-vaccine campaigns were implemented.

References

1. Lewnard JA, Bruxvoort KJ, Fischer H, Hong VX, Grant LR, Jódar L, et al. Prevention of COVID-19 among older adults receiving pneumococcal conjugate vaccine suggests interactions between Streptococcus pneumoniae and SARS-CoV-2 in the respiratory tract. J Infect Dis. 2021. doi:10.1093/infdis/jiab128
2. Patwardhan A, Ohler A. The Flu Vaccination May Have a Protective Effect on the Course of COVID-19 in the Pediatric Population: When Does Severe Acute Respiratory Syndrome Coronavirus 2 (SARS-CoV-2) Meet Influenza? Cureus. 2021;13: e12533.
3. Rivas MN, Ebinger JE, Wu M, Sun N, Braun J, Sobhani K, et al. BCG vaccination history associates with decreased SARS-CoV-2 seroprevalence across a diverse cohort of health care workers. J Clin Invest. 2021;131. doi:10.1172/JCI145157
4. Fernández-Prada M, García-González P, García-Morán A, Ruiz-Álvarez I, Ramas-Diez C, Calvo-Rodríguez C. Personal and vaccination history as factors associated with SARS-CoV-2 infection. Med Clin . 2021;157: 226–233.
