# Supplementary material for: The interactions of SARS-CoV-2 with cocirculating pathogens: Epidemiological implications and current knowledge gaps
Source: PLoS Pathog. 2023 Mar 8;19(3):e1011167. doi: 10.1371/journal.ppat.1011167 (PMC9994710; doi:10.1371/journal.ppat.1011167)
Supplement: S4 Table — (PDF) [file ppat.1011167.s004.pdf]

S4 Table. Observational studies examining the association between prior respiratory infections and COVID-19 [1–4].

Author	Design	Study population	Method to control for confounding	Outcome	Exposure	*Effect Estimate (95% CI or SE)
Kim et al. [1]	Case-control	Individuals covered by national health insurance	Controls matched to cases based on sex, age, income; model adjusted for CCI scores, asthma, COPD, hypertension	COVID-19 diagnosis	Measured in rolling window prior to outcome:<br>a) 1-14 days<br>b) 1-30 days<br>c) 1-90 days<br><br>Prescription of antiviral for influenza treatment<br>URI (ICD-10 J00 to J06)	a) OR=3.07 (1.16, 5.85)<br>b) OR=1.18 (0.72, 1.91)<br>c) OR=1.91 (1.54, 2.37)<br>a) OR=6.95 (6.38, 7.58)<br>b) OR=4.99 (4.64, 5.37)<br>c) OR=2.70 (2.55, 2.86)
				COVID-19 morbidity		a) OR=3.64 (1.55, 9.21)<br>b) OR=3.59 (1.42, 9.05)<br>c) OR=1.54 (0.84, 2.84)<br>a) OR=1.40 (1.11, 1.78)<br>b) OR=1.28 (1.02, 1.61)<br>c) OR=1.17 (0.95, 1.43)
				COVID-19 mortality		a) OR=3.66 (0.71, 18.81)<br>b) OR=3.12 (0.64, 15.19)<br>c) OR=1.62 (0.59, 4.47)<br>a) OR= 0.90 (0.58, 1.40)<br>b) OR= 0.82 (0.54, 1.26)<br>c) OR= 0.77 (0.54, 1.10)

Author	*Design	Study population	Method to control for confounding	Outcome	Exposure	**Effect Estimate (95% CI or SE)
Sager et al. [2]	Cohort-Retrospective	Adults >18yrs whose SARS-CoV-2 PCR result were documented ≥7 days after hCoV PCR result	†Adjusted for age, sex, BMI, DM	PCR+ for SARS-CoV-2 (among tested)	PCR+ for hCoV	OR=0.9 (0.6–1.4)
				COVID-19 hospitalization (among SARS-CoV-2 +)		OR=1.6 (0.8–3.2)
				COVID-19 ICU admission (among hospitalized)		OR=0.1 (0.0–0.7)<br>†OR=0.1 (0.1–0.9)
				COVID-19 MV (among hospitalized)		OR=0.0 (0.0–1.0)
Anderson et al. [3]	Case-control	Individuals whose serum sample were collected before pandemic (2020-03)	Controls matched to cases based on sex, age, race	PCR+ for SARS-CoV-2 (among tested)	hCoV antibodies (OC43 Spike Titer)	***$\beta = 1x10^{-6}$ (SE: $2x10^{-5}$)
				COVID-19 hospitalization (among SARS-CoV-2 +)		***$\beta = 1x10^{-5}$ (SE: $3x10^{-5}$)
				COVID-19 severe hospitalization (among SARS-CoV-2 +)		***$\beta = 2x10^{-5}$ (SE: $5x10^{-5}$)

Author	Design	Study population	Method to control for confounding	Outcome	Exposure	**Effect Estimate (95% CI or SE)
Aran et al. [4]	Cohort-retrospective	Individuals tested for SARS-CoV-2 with ≥12mo enrollment in a private insurance prior to test	Adjusted for sex, age, health-seeking behavior	PCR+ for SARS-CoV-2	Measured within 1 year before study: URI (ICD-10 J01, J02.8/9, J20.9)	OR=0.76 (0.75, 0.77)

Abbreviations CCI: Charlson Comorbidity Index, COPD: chronic obstructive pulmonary disease, URI: upper respiratory infections, HIV: human immunodeficiency virus, BMI: Body Mass Index, DM: Diabetes mellitus, ICU: intensive care unit, MV: mechanical ventilation, ICD: International Classification of Diseases, PCR: Polymerase chain reaction, CI: confidence intervals, OR: odds ratio, SE: standard error.
***Remark 1** Study design was determined to be cohort if participants were selected based on exposure (study compared outcome in exposed vs. unexposed participants) and case-control if participants were selected based on outcome (study compared exposure in participants with vs. without outcome); cohort-prospective means the information on exposure was recorded before the outcome occurred in the study and cohort-retrospective means the information on exposure was recorded after the outcome occurred.
****Remark 2** Effect estimates were OR or β (regression coefficient) directly extracted from studies.
*****Remark 3** β, the regression coefficient, can be interpreted as the increase in ln(odds) for COVID-19 outcome per unit increase in exposure.

References

1. Kim SY, Kim J-H, Kim M, Wee JH, Jung Y, Min C, et al. The associations of previous influenza/upper respiratory infection with COVID-19 susceptibility/morbidity/mortality: a nationwide cohort study in South Korea. Sci Rep. 2021;11: 21568.
2. Sagar M, Reifler K, Rossi M, Miller NS, Sinha P, White LF, et al. Recent endemic coronavirus infection is associated with less-severe COVID-19. J Clin Invest. 2021;131. doi:10.1172/JCI143380
3. Anderson EM, Goodwin EC, Verma A, Arevalo CP, Bolton MJ, Weirick ME, et al. Seasonal human coronavirus antibodies are boosted upon SARS-CoV-2 infection but not associated with protection. doi:10.1101/2020.11.06.20227215
4. Aran D, Beachler DC, Lanes S, Marc Overhage J. Prior Presumed Coronavirus Infection Reduces COVID-19 Risk: A Cohort Study. SSRN Electronic Journal. doi:10.2139/ssrn.3703827
