# Supplementary material for: The interactions of SARS-CoV-2 with cocirculating pathogens: Epidemiological implications and current knowledge gaps
Source: PLoS Pathog. 2023 Mar 8;19(3):e1011167. doi: 10.1371/journal.ppat.1011167 (PMC9994710; doi:10.1371/journal.ppat.1011167)
Supplement: S1 Appendix — (PDF) [file ppat.1011167.s005.pdf]

S1 Appendix. Model details

We developed deterministic compartment models of the two interacting pathogens. Following Shrestha et al. we used a double index notation, e.g $X_{Y,Z}$ where Y gives the state of pathogen 1 and Z the state of pathogen 2 [1].

<u>Bacteria-virus interaction model:</u>

The bacteria-virus model was constructed such that pathogen 1 is the bacteria and pathogen 2 is the virus. We assumed the interaction was asymmetric, such that colonization with bacteria impacts transmission of the virus, but infection with the virus has no impact on the bacterial dynamics. The model was defined by 2 x 3 = 6 ordinary differential equations as represented in Figure S1, where the disease states are {S, C} for bacteria, and {S, I, R} for the virus.

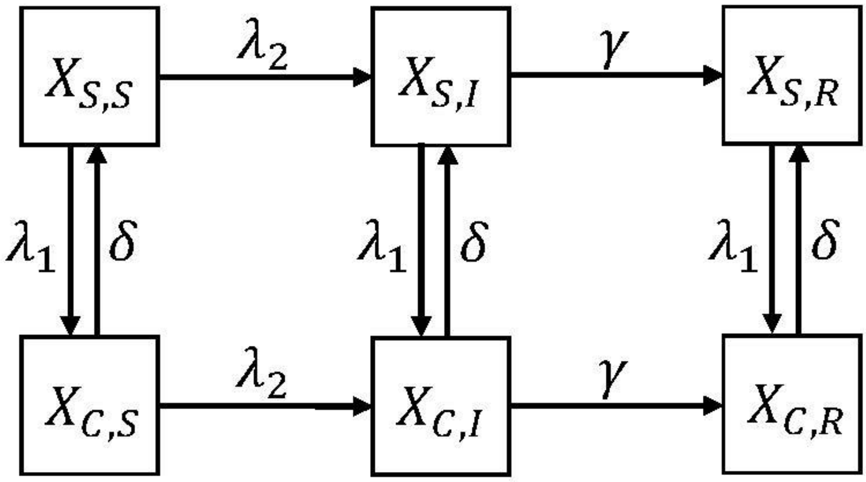

Fig A. Schematic of the bacteria-virus interaction model. Horizontal transitions between compartments are due to virus dynamics, vertical transitions are due to bacteria dynamics.

The forces of infection (λ) and prevalences (p) for each disease are defined as:

$$\lambda_1(t) = (\delta / (1 - C *)) p_1(t)$$

$$p_1(t) = X_{C,S} + X_{C,I} + X_{C,R}$$

$$\lambda_2(t) = R_0 \, \gamma \, p_2(t)$$

$$p_2(t) = X_{S,I} + \theta \, X_{C,I}.$$

Where $1/\delta$ is the average period of bacteria colonization, $C^* = \lim_{t \to \infty}(X_{C,S} + X_{C,I} + X_{C,R})$ is the endemic colonization prevalence. $R_0$is the initial reproduction number of the virus, and $1/\gamma$ is the average recovery period of the virus. θ is an interaction parameter indicating the impact of bacterial carriage on transmission.

As an example we consider the *S. pneumoniae* - SARS-CoV-2 interacting system, hence assuming the bacteria is *S. pneumoniae* and the virus is SARS-CoV-2. Parameter values are detailed in Table S5. The model was run for 365 days and the peak viral incidence was calculated for varying rate of bacterial colonization and varying transmission interaction parameter. The model was implemented in the R [2] packages 'pomp' [3], and 'tidyverse' [4]. Plots were created with 'ggplot2' [5], 'patchwork' [6], 'scico' [7] and 'Microsoft PowerPoint'. All code is available at https://github.com/egoult/pathogen_coinfections .

Table A. Parameters used for *S. pneumoniae* - SARS-CoV-2 interaction model.

Parameter	Meaning	Fixed values	Source
C^*	Bacterial colonization prevalence	0–0.6	[8,9]

Parameter	Meaning	Fixed values	Source
$1/\delta$	Duration of bacterial colonization	50 days	[10,11]
I_0	Initial fraction infected with virus	1 x 10^{-5}	Assumption
R_0	Virus basic reproductive number	2	[12]
$1/\gamma$	Virus recovery period	9 days	[12,13]
θ	Coinfection impact on viral transmission	0.2, 0.5, 0.8, 1.0, 1.2, 2.0, 5.0	Assumption

Virus-virus interaction model

The virus virus interaction model was also constructed asymmetrically, so infection with virus 1 impacts transmission of virus 2, but infection with virus 2 has no impact on virus 1. The model is defined in 4 x 4 = 16 ordinary differential equations as shown in Figure S2 with each virus having the disease states {SEIR} [14].

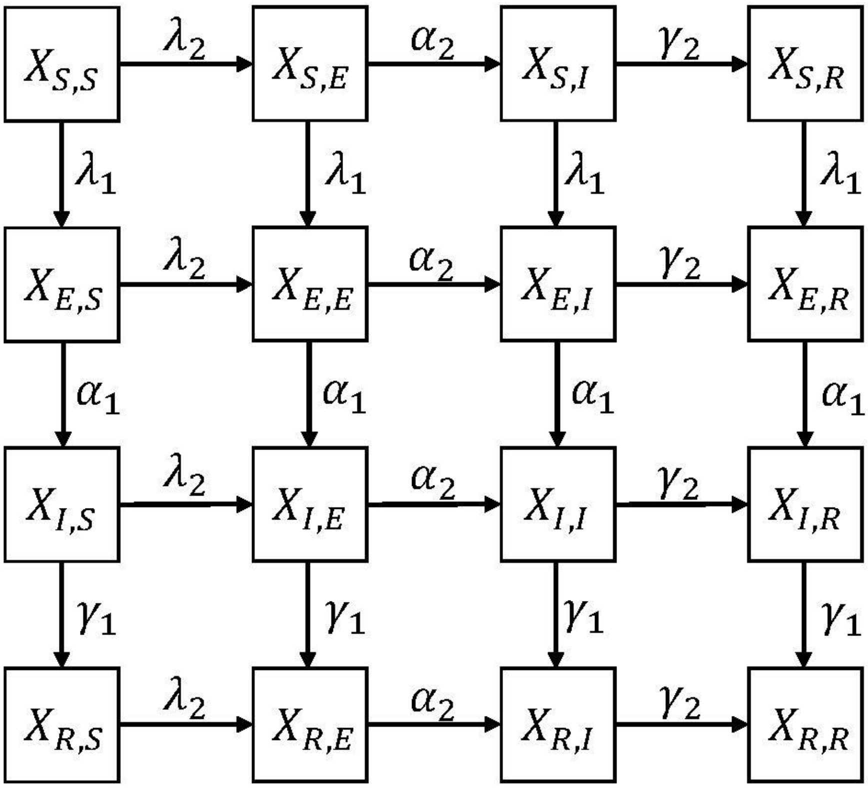

Fig B. Schematic of the virus virus interactions model. Horizontal transitions are due to virus 2 dynamics, and vertical transitions due to virus 1 dynamics.

The forces of infection (λ) and prevelances (p) of each virus are defined as:

$$\lambda_1(t) = R_{0,1}\gamma_1 p_1(t)$$

$$p_1(t) = X_{I,S} + X_{I,E} + X_{I,I} + X_{I,R}$$

$$\lambda_2(t) = R_{0,2}\gamma_2 p_2(t)$$

$$p_2(t) = X_{S,I} + X_{E,I} + \theta X_{I,I} + X_{R,I}$$

Here, $R_{0,1}$ and $R_{0,2}$ denote the respective basic reproductive numbers for virus 1 and virus 2. $1/\alpha_1$ and $1/\alpha_2$ are the respective incubation periods and $1/\gamma_1$ and $1/\gamma_2$ the recovery periods for the viral diseases. The parameter θ is an interaction parameter indicating the impact of infection with virus 1 on transmission of virus 2.

We consider the Influenza A - SARS-CoV-2 interacting system as an example, where virus 1 is Influenza A and virus 2 is SARS-CoV-2, so infection with influenza A affects the dynamics of SARS-CoV-2, but infection with SARS-CoV-2 has no impact on influenza A. Parameter values are detailed in Table S6. The model was run for 365 days and the peak SARS-Cov-2 incidence was calculated, for varying of influenza A basic reproduction numbers and varying transmission interaction parameter.

Table B. Parameters used for influenza A - SARS-CoV-2 interaction model.

Parameter	Meaning	Fixed values	Source
$X_{E,S}(t = 0)$	Initial fraction exposed to virus 1	1×10^{-3}	Assumption
$X_{R,S}(t = 0)$	Initial fraction immune to virus 1	0.2	Assumption
$R_{0,1}$	Virus 1 basic reproductive number	1.0–2.5	[15]
$1/\alpha_1$	Virus 1 latent period	1 day	[16]
$1/\gamma_1$	Virus 1 recovery period	4 days	[17]
$X_{S,E}(t = 0)$	Initial fraction exposed to virus 2	1×10^{-5}	Assumption

Parameter	Meaning	Fixed values	Source
$R_{0,2}$	Virus 2 basic reproductive number	2	[12]
$1/\alpha_2$	Virus 2 latent period	4 days	[12]
$1/\gamma_2$	Virus 2 recovery period	5 days	[13]
θ	Coinfection impact on viral transmission	0.2, 0.5, 0.8, 1.0, 1.2, 2.0, 5.0	Assumption

References

1. Shrestha S, King AA, Rohani P. Statistical inference for multi-pathogen systems. PLoS Comput Biol. 2011;7: e1002135.
2. R Core Team. R: The R Project for Statistical Computing. Accessed August 11, 2021. https://www.r-project.org/
3. King AA, Nguyen D, Ionides EL. Statistical Inference for Partially Observed Markov Processes via the R Package pomp. *J Stat Softw*. 2016;69:1-43. doi:10.18637/JSS.V069.I12
4. Wickham H, Averick M, Bryan J, et al. Welcome to the Tidyverse. *J Open Source Softw*. 2019;4(43):1686. doi:10.21105/JOSS.01686
5. Wickham H. *Ggplot2 Elegant Graphics for Data Analysis*. Second Edition. Springer Nature; 2016. Accessed April 11, 2022. http://www.springer.com/series/6991
6. Thomas M, Pedersen L. Package "patchwork" Type Package Title The Composer of Plots Version 1.1.1. Published online 2020.
7. Thomas M, Pedersen L. Package "scico" Title Colour Palettes Based on the Scientific Colour-Maps. Published online 2021. Accessed April 11, 2022. https://github.com/thomasp85/scico/issues
8. Hussain M, Melegaro A, Pebody RG, George R, Edmunds WJ, Talukdar R, et al. A longitudinal household study of *Streptococcus pneumoniae* nasopharyngeal carriage in a UK setting. Epidemiology and Infection. 2005. pp. 891–898. doi:10.1017/s0950268805004012
9. Regev-Yochay G, Raz M, Dagan R, Porat N, Shainberg B, Pinco E, et al. Nasopharyngeal carriage of Streptococcus pneumoniae by adults and children in community and family settings. Clin Infect Dis. 2004;38: 632–639.
10. Almeida ST, Paulo AC, Froes F, de Lencastre H, Sá-Leão R. Dynamics of Pneumococcal Carriage in Adults: A New Look at an Old Paradigm. The Journal of Infectious Diseases.

2021. pp. 1590–1600. doi:10.1093/infdis/jiaa558

11. Dube FS, Ramjith J, Gardner-Lubbe S, Nduru P, Lourens Robberts FJ, Wolter N, et al. Longitudinal characterization of nasopharyngeal colonization with Streptococcus pneumoniae in a South African birth cohort post 13-valent pneumococcal conjugate vaccine implementation. Scientific Reports. 2018. doi:10.1038/s41598-018-30345-5
12. Li R, Pei S, Chen B, Song Y, Zhang T, Yang W, et al. Substantial undocumented infection facilitates the rapid dissemination of novel coronavirus (SARS-CoV-2). Science. 2020;368: 489–493.
13. Bi Q, Wu Y, Mei S, Ye C, Zou X, Zhang Z, et al. Epidemiology and transmission of COVID-19 in 391 cases and 1286 of their close contacts in Shenzhen, China: a retrospective cohort study. The Lancet Infectious Diseases. 2020. pp. 911–919. doi:10.1016/s1473-3099(20)30287-5
14. Domenech de Cellès M, Goult E, Casalegno J-S, Kramer SC. The pitfalls of inferring virus–virus interactions from co-detection prevalence data: application to influenza and SARS-CoV-2. Proceedings of the Royal Society B: Biological Sciences. 2022. doi:10.1098/rspb.2021.2358
15. Biggerstaff M, Cauchemez S, Reed C, Gambhir M, Finelli L. Estimates of the reproduction number for seasonal, pandemic, and zoonotic influenza: a systematic review of the literature. BMC Infect Dis. 2014;14: 480.
16. Carrat F, Vergu E, Ferguson NM, Lemaitre M, Cauchemez S, Leach S, et al. Time lines of infection and disease in human influenza: a review of volunteer challenge studies. Am J Epidemiol. 2008;167: 775–785.
17. Vink MA, Bootsma MCJ, Wallinga J. Serial intervals of respiratory infectious diseases: a systematic review and analysis. Am J Epidemiol. 2014;180: 865–875.
